# Intraclass correlation – A discussion and demonstration of basic features

**DOI:** 10.1371/journal.pone.0219854

**Published:** 2019-07-22

**Authors:** David Liljequist, Britt Elfving, Kirsti Skavberg Roaldsen

**Affiliations:** 1 Department of Physics, Stockholm University, Stockholm, Sweden; 2 Department of Neurobiology, Care Science and Society, Division of Physiotherapy, Karolinska Institutet, Stockholm, Sweden; 3 Department of Research, Sunnaas Rehabilitation Hospital, Nesodden, Norway; 4 Faculty of Health Sciences, Department of Physiotherapy, Oslo Metropolitan University, Oslo, Norway; University of Catania, ITALY

## Abstract

A re-analysis of intraclass correlation (ICC) theory is presented together with Monte Carlo simulations of ICC probability distributions. A partly revised and simplified theory of the single-score ICC is obtained, together with an alternative and simple recipe for its use in reliability studies. Our main, practical conclusion is that in the analysis of a reliability study it is neither necessary nor convenient to start from an initial choice of a specified statistical model. Rather, one may impartially use all three single-score ICC formulas. A near equality of the three ICC values indicates the absence of bias (systematic error), in which case the classical (one-way random) ICC may be used. A consistency ICC larger than absolute agreement ICC indicates the presence of non-negligible bias; if so, classical ICC is invalid and misleading. An F-test may be used to confirm whether biases are present. From the resulting model (without or with bias) variances and confidence intervals may then be calculated. In presence of bias, both absolute agreement ICC and consistency ICC should be reported, since they give different and complementary information about the reliability of the method. A clinical example with data from the literature is given.

## 1. Introduction

The intra-class correlation coefficient (ICC) is a number, usually found to have a value between 0 and 1. It is a well-known statistical tool, applied for example in medical, psychological, biological and genetic research. It refers to correlations within a class of data (for example correlations within repeated measurements of weight), rather than to correlations between two different classes of data (for example the correlation between weight and length). There are several different versions of the ICC; the choice of the proper version depends on the experimental situation.

The ICC is recommended as one measure (among others) of the reliability of an experimental method [1]. It is sensitive for example to the extent to which subjects (individuals) keep their ranking order in repeated measurements. Moreover, it may indicate the ability of an experimental method to detect and measure systematic differences between subjects. This ability is limited since those differences may be more or less masked by individual variations of random nature within subjects and by errors (uncertainties) of random nature and biases (systematic errors) in the method of measurement. For the method to be reliable and useful, such unwanted variations have to be sufficiently small. The ICC serves as a quantitative estimate of this aspect of reliability.

Very generally speaking, the ICC is calculated as a ratio ICC = (variance of interest) / (total variance) = (variance of interest) / (variance of interest + unwanted variance). If the unwanted variance is equal to or larger than the variance of interest (for example, the variance between subjects) the reliability of the method is evidently poor; as can be seen, the ICC has then a value below 0.5. On the other hand, ICC values above 0.8 or 0.9 are often regarded as a sign of good or excellent reliability [2]. However, whether an ICC value is good enough should depend on the intended use of the method.

The ICC concept was originally presented by Fisher [3]. Present studies of reliability in for example medical (clinical) research usually refer, directly or indirectly, to the theory developed by Bartko [4], Shrout and Fleiss [5] and McGraw and Wong [6] (see, e.g., references [7–16] for recent work). It may be noted that this theory is based on the analysis of variance (ANOVA), assuming normal (Gaussian) distributions. Other approaches to the calculation of variances and ICC exist, using for example maximum likelihood methods [17,18] and addressing the calculation of ICC also for non-Gaussian data [18]. However, we will here restrict ourselves to the ANOVA-based approach which, as mentioned, is the one usually applied in medical research.

Despite its widespread use, the meaning and proper application of the ICC may present a problem to the non-statistician researcher. The recipes for choosing the appropriately specified statistical model and ICC formula are not always easily interpreted, i.e. applied to the experimental situation at hand. Indeed, it seems possible that ICC statistical software may appear to the non-statistician user as a "black box", where the user's task essentially is to decipher the verbal instructions for how to make the choices that lead to the hopefully correctly specified statistical model and the corresponding ICC formula.

Our purpose with this article is to present a revised and simplified theory of the single-score ICC and an alternative recipe for its use. The main application that we have in mind is reliability studies in medical (clinical) research.

For this purpose we have performed a re-analysis of the theory presented by refs. [4–6] complemented by demonstrations using Monte Carlo simulation. We limit the present discussion to single-score ICC; i.e., each measured value used in the analysis represents a single measurement, not the average of two or more measurements. In the notation used by McGraw and Wong [6] this results in three distinct ICC formulas, named ICC(1) (the original ICC without bias, introduced by Fisher [3]), ICC(A,1) (absolute agreement ICC in the presence of bias) and ICC(C,1) (consistency ICC in the presence of bias). These three formulas are identical to the three formulas ICC(1,1), ICC(2,1) and ICC(3,1), so named and discussed by Shrout and Fleiss [5]; however, the notation used by McGraw and Wong is clarifying by systematically separating the absolute agreement (A) and consistency (C) versions of the ICC; therefore, we have chosen to follow their notation.

We address that question which has been discussed in a considerable number of papers, namely: which statistical model is the correct one? Which of these three ICC formulas should be chosen? Previous discussions are in general directly or indirectly based on the verbal recommendations presented by Shrout and Fleiss [5], quoting these recommendations more or less literally.

However, these recommendations are formulated with one rather special situation in mind, namely raters judging targets (subjects) [5]. This formulation might not be easily and unambiguously translated to other experimental situations. In repeated clinical measurements, biases associated with the measuring equipment or the arrangement of successive tests may be more important than the identity of the person performing the measurement.

Moreover, the approach presented by references [4–6] emphasizes the importance of a specified statistical model appropriate for the experimental design. This has led to the use of flow charts [2,6] for helping the researcher to identify the appropriate model and the correct ICC formula. Such a flow chart is for example implied in the well-known statistical package SPSS. On basis of our re-analysis and Monte Carlo simulation we will however propose a different and simpler procedure, where an initial choice of a specified model is not required for the analysis of experimental data. A similar recommendation has previously been given by Weir [19].

The remainder of the article is organized as follows. In Section 2 we revisit the ICC theory described by refs. [4–6]. Aiming at transparency we present a thorough discussion and re-analysis of basic derivations and concepts, i.e. the one-way random, the two-way random and the two-way mixed statistical model.

In Section 3, we briefly describe the principles of the present simulation procedures. In Section 4 we perform simulations of the probability distributions of the ICC values generated by the three statistical models, with the purpose of presenting a general and graphical overview of the properties of the single-score ICC.

In Section 5 we present an alternative recipe for the practical evaluation of ICC in a reliability study. Section 6 is concerned with the strategy of analysis and with a demonstration of the equivalence of the two-way random and two-way mixed models in reliability studies. Section 7 summarizes briefly our main conclusion as regards the practical calculation of ICC and of how it may be reported.

Symbols and abbreviations frequently used in this article are shown in Table 1.

**Table 1:** Symbols and abbreviations.

Model 1 = one-way random model (no bias) Model 2 = two-way random model (random bias) Model 3 = two-way mixed model (fixed bias) *n* = number of subjects (targets) *k* = number of measurements (conditions, raters) *i* = subject index (*i* = 1,…, *n*) *j* = measurement index (*j* = 1,…, *k*) *N* = number of simulated matrices < … > = average value obtained in simulation *μ* = population mean of subject's scores *r*_*i*_ = deviation from mean for subject *i* *c*_*j*_ = bias in measurement *j* *v*_*ij*_ = "noise" = error in measurement *j* for subject *i*, Model 1 *e*_*ij*_ = error in measurement *j* for subject *i*, Model 2 and Model 3 *rc*_*ij*_ = interaction in measurement *j* for subject *i*, Model 2 and Model 3 *v*_*ij*_ = "noise" = *e*_*ij*_ + *rc*_*ij*_ (Model 2 and Model 3) *σ*_*r*_^2^ = variance of *r*_*i*_ *σ*_*c*_^2^ = variance of *c*_*j*_ *σ*_*e*_^2^ = variance of *e*_*ij*_ *σ*_*rc*_^2^ = variance of *rc*_*ij*_ *σ*_*v*_^2^ = variance of *v*_*ij*_ (= *σ*_*rc*_^2^ + *σ*_*e*_^2^ in Model 2 and Model 3) *ρ*_1_ = population ICC, Model 1 *ρ*_2A_ = absolute agreement population ICC, Model 2 *ρ*_2C_ = consistency population ICC, Model 2 *ρ*_3A_ = absolute agreement population ICC, Model 3 *ρ*_3C_ = consistency population ICC, Model 3 *MST* = mean square total *MSWS* = mean square within subjects *MSBS* = mean square between subjects *MSWM* = mean square within measurements *MSBM* = mean square between measurements *MSE* = mean square error *F* = *MSBM/MSE* = F-value ICC(1) ≡ ICC(1,1) = sample ICC formula, Model 1 ICC(A,1) ≡ ICC(2,1) = sample ICC formula, absolute agreement, Model 2 and 3 ICC(C,1) ≡ ICC(3,1) = sample ICC formula, consistency, Model 2 and 3

## 2. Method, Part I: Theory

### 2.1. Matrix of measured data

The intra-class correlation has been used to characterize a wide range of different experimental data. It has been applied for example to directly measured physical data, such as muscular strength [20] and force [21]; to rated time of physical activity [22] and to reported subjective data such as sum scores of pain and anxiety symptoms [23]. We will refer to all such data as the result of measurements. One important restriction is that the result of a measurement is a quantity which can be represented in a meaningful way by a real number, i.e. by numerical data rather than categorical data [24]. A general description which covers most experimental situations of interest, provided that the ICC is applicable, should then be the following.

From a population *P*, a number *n* of subjects (*i* = 1,2,…,*n*) have been randomly selected. On these subjects some kind of measurement of a specific quantity *x* (for example, blood pressure) is performed. On each subject, the measurement is made *k* times (*j* = 1,2,…,*k*). The result obtained for subject (*i*) in measurement (*j*) is a real number *x*_*ij*_ (a blood pressure value). The complete experimental result for all the subjects and all the measurements may thus be written [4] as a matrix with *n* rows and *k* columns, i.e., a rectangular arrangement of numbers *x*_*ij*_ such as shown in Table 2. The "subjects" are almost always assumed to be individuals (in the literature sometimes referred to as, e.g., "targets" or "people"), and the "measurements" are often referred to as for example "conditions" or "raters", depending on the experimental or clinical situation.

**Table 2:** Experimental data matrix with *n* rows and *k* columns.

Subject	Measurement 1	Measurement 2	…	Measurement *k*	Mean
1	*x*_*11*_	*x*_*12*_	…	*x*_*1k*_	*S*_*1*_
2	*x*_*21*_	*x*_*22*_	…	*x*_*2k*_	*S*_*2*_
…	…	…	…	…	…
…	…	…	…	…	…
*n*	*x*_*n1*_	*x*_*n2*_	…	*x*_*nk*_	*S*_*n*_
**Mean**	*M*_*1*_	*M*_*2*_	…	*M*_*k*_	$\bar{x}$

Each row contains the data from one particular subject (target), while each column contains the data from one particular measurement (rater, condition). From the measured data *x*_*ij*_ we calculate the mean value *S*_*i*_ for each row, the mean value *M*_*j*_ for each column and the total mean value $\bar{x}$ of all *x*_*ij*_.

We now wish to calculate an ICC value from this sample of *n* × *k* measured data. This sample ICC value is a quantity that characterizes this particular set of data, but, more interestingly, it is also a statistical estimate of the intra-class correlation coefficient *ρ* which characterizes the method of measurement more generally, i.e. when it is applied to any randomly selected sample of subjects from the population *P*. Thus, the ICC value may tell something about the reliability of the method.

### 2.2. Analysis of variance

Regardless of which ICC version is to be used, we first need to perform ANOVA (Analysis of Variance) on the measured data matrix, calculating the various possible sums of squares (*SS*) and, from them, the mean squares (*MS*). The experimental situation described in section 2.1 motivates the use of the so-called repeated measures ANOVA [1]. We do not yet have to consider which of these *MS* we actually will need when calculating the desired ICC; the *SS* and *MS* are all easily calculated anyhow, and for clearness we list their definitions in Appendix 1 (S1 Appendix), as well as the most important relations among them. In so doing we also define the presently used notation. We prefer a four-letter notation (*MSWS* = Mean Square Within Subjects; *MSBS* = Mean Square Between Subjects; *MSBM* = Mean Square Between Measurements; *MSWM* = Mean Square Within Measurements) except for *MSE* (= Mean Square, Error) and *MST* (= Mean Square, Total). This notation seems to be more unambiguous and also easier to interpret and memorize than the somewhat varying three-letter notations that are more commonly used.

From now on we assume that all the required mean squares have been calculated from a measured data matrix such as indicated in Table 2.

### 2.3. Model 1, leading to formula ICC(1)

#### 2.3.1. Statistical model and population ICC

It cannot be too much emphasized that what underlies each ICC formula is a specific mathematical model of the experimental situation. For an ICC formula to be valid and appropriate, it is required that this model is a reasonably realistic description of the actual situation.

The first model to be discussed here is sometimes referred to as the Case 1 model [5,6], sometimes as the one-way random effect model [6], or, more briefly, the one-way model. The name refers to the fact that a random sampling of subjects is assumed. It is also sometimes known as a linear or linear mixed-effects model (LMM) [18]. It was presented already in the early half of the twentieth century by Fisher [3] and leads directly to the classical ICC formula sometimes known as ICC(1,1) [5] or as ICC(1) [6]. We will simply refer to it as Model 1.

We may here dwell for a moment on terminology. Model 2, to be discussed later, is referred to as a two-way random effects model, meaning that a random sampling of measurement biases are introduced as well. (Note: this should not be confused with one-way and two-way ANOVA.) Model 3, finally, is described as a two-way mixed model [6], meaning that a random sampling of subjects is again assumed, while the biases are assumed to be fixed.

In statistical theory it is often assumed that a measured value may be regarded as the sum of a "true score" and an "error". In Model 1 it is specifically assumed that each experimental value *x*_*ij*_ in the matrix of measured data indicated in Table 2 may be regarded as a sum of three contributions:

$$x_{ij}=\mu +r_{i}+v_{ij}$$ (1)

In Eq (1), *μ* is a constant, namely the mean value of *x* for the whole population *P*, while *μ* + *r*_*i*_ is the "true score" for subject *i*, i.e. that value which would be obtained for subject *i* if there was no error in the measurement. When a subject (*i*) is selected from the population *P*, it is assumed that the *r*_*i*_ value is sampled (randomly picked) from a normal distribution with mean value = 0 and standard deviation σ_*r*_. In other words, it is assumed that the "true scores" are distributed among the individuals in the population *P* in accordance with a normal distribution with mean value *μ* and standard deviation σ_*r*_.

The third term *v*_*ij*_ is often called "error". A non-zero value may indeed be due to an error in the measurement, but it may also be, for example, a physiologically caused variation of the *x* value from its "true score" *μ* + *r*_*i*_ at the time of the measurement. For each *x*_*ij*_ value, a separate *v*_*ij*_ value is assumed to be sampled from a normal distribution with a mean value = 0 and a standard deviation *σ*_*v*_. This means that the error contributions *v*_*ij*_ to different matrix elements *x*_*ij*_ are independent of each other; it also means that the average value of *x*_*ij*_ over a large number of measurements will be *μ* + *r*_*i*_. The term *v*_*ij*_ is conventionally written *w*_*ij*_ [5,6]; the reason for using *v*_*ij*_ in the present context will be obvious when Model 2 is discussed in the context of Eq (8). It may be noted that Eq (1), specifies exactly how to simulate the ICC(1) intraclass correlation coefficient; the details of this are described in Section 3.

By means of a simple example we can, following Fisher [3], see that the concept of an intraclass correlation, i.e., a correlation *within* the measured data *x*_*ij*_, now arises naturally. Assume, for example, that *k* = 2 measurements have been made on each of *n* subjects. In a diagram with values from the first measurement (*x*_*i*1_, *i* = 1,2, …*n*) on the horizontal axis and values from the second measurement (*x*_*i*2_, *i* = 1,2, …*n*) on the vertical axis, the two measurements on each subject will be represented by one point in the diagram. Obviously, the result (a scatter plot with *n* points) is analogous to a correlation such as described by the more well-known interclass (Pearson) correlation coefficient *r*. If the average magnitude (≈ *σ*_*v*_) of the error *v*_*ij*_ is small compared to the average magnitude (≈ *σ*_*r*_) of the differences between different true scores *μ* + *r*_*i*_ for the individuals, the points will be well spread out along and near the line of equality; this is a case of high intraclass correlation. With a larger number of measurements (*k* > 2) a high intraclass correlation (without bias) means that the points will be spread out along the line of equality in a *k*-dimensional space.

Accordingly, Fisher [3] introduced an intraclass correlation coefficient formula analogous in form to the interclass (Pearson) correlation coefficient *r*. However, if the number of subjects *n* is very large (properly speaking, infinite), then it turns out that the Model 1 intraclass correlation coefficient is simply given by [3]

$$\rho_{1}=\frac{\sigma_{r}{}^{2}}{\sigma_{r}{}^{2}+\sigma_{v}{}^{2}}$$ (2)

i.e. the variance of the true score among the subjects in the population *P*, divided by the total variance.

This coefficient *ρ*_1_ may be regarded as the intraclass coefficient of the method when applied to the entire (and presumably very large) population *P*; consequently, it is sometimes referred to as the population ICC [6]. As is obvious from Eq (2), *ρ*_1_ may have values ranging from arbitrarily near 0 (low or non-existent reliability, *σ*_*v*_ >> *σ*_*r*_) to arbitrarily near 1 (high reliability; *σ*_*v*_ << *σ*_*r*_).

#### 2.3.2. Expected mean square relations. The ICC(1) formula

We wish to estimate the standard deviations *σ*_*v*_, *σ*_*r*_ and thus also the population intraclass coefficient *ρ*_1_ from a limited sample of subjects randomly selected from the population *P*, i.e. from a single experimental matrix such as indicated in Table 2. For this we need the ANOVA relations giving the so-called expected mean squares (EMS).

The EMS relations are of key importance for understanding the intraclass correlation formulas, but in medical and psychological articles as well as in medical statistics textbooks they are seldom quoted, then only partly, without any derivation and even without giving any references [1,5,6]. For this reason, and also since we will use them extensively, we have included a simple but comprehensive derivation of the EMS relations in Appendix 2 (S2 Appendix), for Model 1, Model 2 and the presently considered modified version of Model 3.

Using the EMS relations for Model 1 (see S2 Appendix) we obtain statistical estimates of the variances *σ*_*r*_^2^ and *σ*_*v*_^2^ from a sample of *n × k* experimental data *x*_*ij*_ such as indicated in Table 2. For example, it is found that the mean square quantity *MSBS* (Mean Square Between Subjects) is in fact a statistical estimate of *k*·*σ*_*r*_^2^ + *σ*_*v*_^2^, while *MSWS* (Mean Square Within Subjects) is an estimate of *σ*_*v*_^2^. In other words, an approximate equality is expected, so we may write these two EMS relations as

$$\begin{array}{l} MSBS\approx k\cdot \sigma_{r}{}^{2}+\sigma_{v}{}^{2}\\ MSWS\approx \sigma_{v}{}^{2} \end{array}$$ (3)

The derivation of the EMS relations (S2 Appendix) as well as the simulations described in Section 4 demonstrate that the exact meaning of Eq (3) is that if the entire experiment (*n* subjects, each subjected to *k* measurements) is repeated an infinite number of times, then the resulting average values of *MSBS* and *MSWS*, denoted <*MSBS*> and <*MSWS*>, will be exactly equal to the right members in Eq (3), i.e.

$$\begin{array}{l} <MSBS>=k\cdot \sigma_{r}{}^{2}+\sigma_{v}{}^{2}\\ <MSWS>=\sigma_{v}{}^{2} \end{array}$$ (4)

We cannot perform the experiment an infinite number of times, but when values of *MSBS* and *MSWS* are calculated from one single experimental data matrix, they are most probably found near these average values. Thus, Eq (3) is justified. Solving Eq (3) algebraically for the *σ*:s we have

$$\begin{array}{l} \sigma_{r}{}^{2}\approx \frac{MSBS-MSWS}{k}\\ \sigma_{v}{}^{2}\approx MSWS \end{array}$$ (5)

Evidently, Eq (5) provides us with the desired estimates of the variances *σ*_*r*_^2^ and *σ*_*v*_^2^. Moreover, using Eq (5) in Eq (2), we get

$$\rho_{1}=\frac{\sigma_{r}{}^{2}}{\sigma_{r}{}^{2}+\sigma_{v}{}^{2}}\approx \frac{MSBS-MSWS}{MSBS+(k-1)\cdot MSWS}\equiv ICC(1)$$ (6)

In the last two members of Eq (6) we have obtained the well-known standard form of the sample ICC formula ICC(1), written in terms of the mean squares *MSBS* and *MSWS*. Thus, the sample value ICC(1), calculated from the *MSBS* and *MSWS* values, may be regarded as an estimate of the population intra-class correlation *ρ*_1_ defined by Eq (2).

We may note one detail previously discussed e.g. by Lahey *et al*. [25]. The expression for ICC(1) in terms of the mean squares *MSBS* and *MSWS* may become negative, namely if *MSBS < MSWS*. Now, according to Eq (2), *ρ*_1_ cannot be negative, and if *MSBS* and *MSWS* are good estimates, then we should, as seen from Eq (3), expect *MSBS > MSWS*. Consequently, a negative ICC(1) is simply a bad (unfortunate) estimate. This may occur by chance, especially if the sample size (number of subjects *n*) is small.

### 2.4. Model 2, leading to formulas ICC(A,1) and ICC(C,1)

#### 2.4.1. Statistical model and population ICC

Conventionally, Model 2 (the two-way random model) is specified by the following statistical model [4–6]. Each value *x*_*ij*_ in the experimental data matrix is assumed to be the sum of five terms:

$$x_{ij}=\mu +r_{i}+c_{j}+rc_{ij}+e_{ij}$$ (7)

The symbols *μ* and *r*_*i*_ have the same meaning as in Model 1. Thus, the *r*_*i*_ term is sampled from a normal distribution with mean = 0 and standard deviation *σ*_*r*_. However, one new term *c*_*j*_ is added. Moreover, the term *v*_*ij*_ that describes the random fluctuations in each measurement has been replaced by the sum of two different contributions *rc*_*ij*_ and *e*_*ij*_.

The term *c*_*j*_ represents a systematic error (bias), common to all measurements labeled (*j*); i.e. it affects column (*j*) in the data matrix. For example, these measurements may have been performed by a rater, who, for some reason, has a tendency to obtain somewhat higher *x*_*ij*_ values. Following Bartko [26], who named *c*_*j*_ an "additive bias", we will refer to the *c*_*j*_ terms simply as "bias terms". In Model 2 it is assumed that these bias terms are random, in the sense that each *c*_*j*_ value is sampled from a normal distribution with a mean value = 0 and standard deviation *σ*_*c*_; thus, if the entire experiment is repeated with *k* measurements on each of *n* new subjects, then the biases *c*_*j*_ will in general not be the same. For example, raters may have been replaced by new raters with other biases.

The term *rc*_*ij*_ is a so-called interaction term; it takes into account that the effect of bias may not be the same for all subjects. In Model 2 it is assumed that for each *x*_*ij*_, the value of *rc*_*ij*_ is to be sampled from a normal distribution with mean = 0 and standard deviation *σ*_*rc*_. The term *e*_*ij*_, finally, is a residual (error) term. It is, for each *x*_*ij*_ separately, sampled from a normal distribution with mean = 0 and standard deviation *σ*_*e*_.

We wish to stress the fact that these verbal descriptions of the various terms should be regarded as a statistical interpretation of the model given by Eq (7). What defines the model from a purely mathematical point of view is the algebraic form of Eq (7) and the precise specifications of how the various terms are to be sampled from various normal distributions.

Therefore, we may use the well-known mathematical fact that if a value *rc*_*ij*_ is sampled from a normal distribution with mean = 0 and variance *σ*_*rc*_^2^, and another value *e*_*ij*_ is sampled from another normal distribution with mean = 0 and variance *σ*_*e*_^2^, and they are added as in Eq (7), then the result is equivalent to sampling one single value *v*_*ij*_ from a normal distribution with mean = 0 and variance *σ*_*v*_^2^ = *σ*_*rc*_^2^ + *σ*_*e*_^2^. Consequently, we may, with the above sampling prescriptions, quite generally write the statistical model Eq (7) more simply as

$$x_{ij}=\mu +r_{i}+c_{j}+v_{ij}$$ (8)

where *v*_*ij*_ is sampled from a normal distribution with mean = 0 and standard deviation *σ*_*v*_. The *rc*_*ij*_ and *e*_*ij*_ terms have been merged into one single term *v*_*ij*_. The meaning of the *v*_*ij*_ term is mathematically precisely the same in Model 1 and Model 2, namely a term whose value is sampled from a normal distribution with a standard deviation *σ*_*v*_. Comparison of Eq (8) and Eq (1) then shows that the only thing that differs Model 2 from Model 1 is the presence of the bias terms *c*_*j*_.

The population intraclass correlation coefficient corresponding to the model given by Eq (8) is again given by the variance of interest divided by the total variance, i.e. [4]

$$\rho_{2A}=\frac{\sigma_{r}{}^{2}}{\sigma_{r}{}^{2}+\sigma_{c}{}^{2}+\sigma_{v}{}^{2}}$$ (9)

This population intraclass coefficent is said to describe the "absolute agreement" between different measurements [6]; hence the "*A*", which here appears as an index in *ρ*_*2A*_. The meaning of this will be more apparent when Eq (9) is compared with the "consistency" intraclass correlation defined below.

In order to obtain a statistical estimate of *ρ*_*2A*_, the EMS relations for Model 2 are needed. They are (see S2 Appendix)

$$\begin{array}{l} MSBS\approx k\cdot \sigma_{r}{}^{2}+\sigma_{v}{}^{2}\\ MSBM\approx n\cdot \sigma_{c}{}^{2}+\sigma_{v}{}^{2}\\ MSWS\approx \sigma_{c}{}^{2}+\sigma_{v}{}^{2}\\ MSWM\approx \sigma_{r}{}^{2}+\sigma_{v}{}^{2}\\ MSE\approx \sigma_{v}{}^{2} \end{array}$$ (10)

where we have, for completeness and later convenience, included the expected mean square *MSWM* (Mean Square Within Measurements).

Also for convenience we will henceforward refer to the *v*_*ij*_ terms as "noise" terms; regardless of their origin, they are (roughly) analogous to what is known in signal theory as “noise”, i.e. an in general unwanted random variation that tends to drown the signal (the true scores). For example, a high ICC(1) value corresponds to a high signal-to-noise ratio.

**Fig 1:**
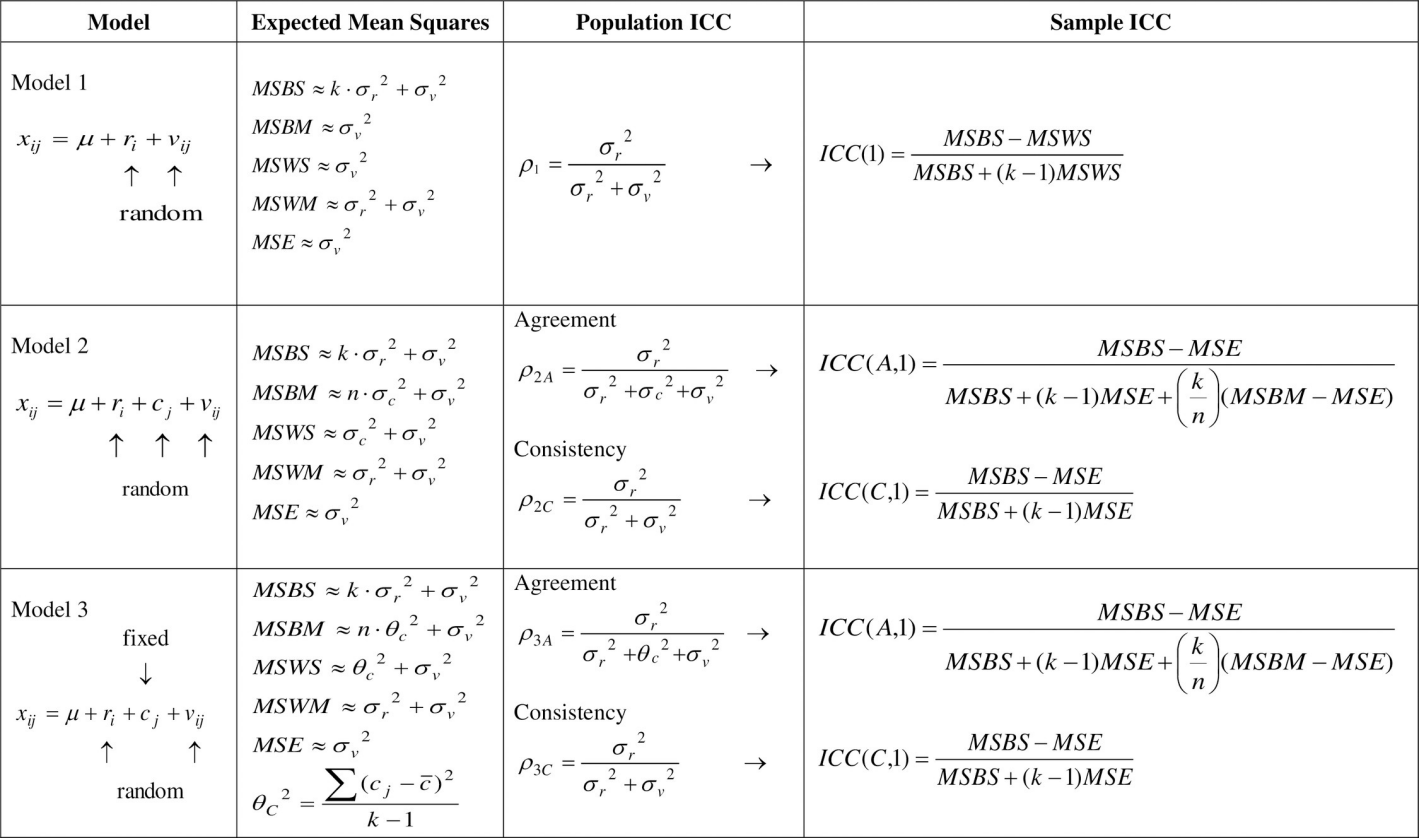
Survey of models, mean square relations and ICC formulas.

The complete set of EMS relations for Model 1 (including, as a detail, Eq (3)) are obtained from Eq (10) simply by putting *σ*_*c*_^2^ = 0. This is also seen in Fig 1, where all models, EMS relations and ICC formulas discussed here are summarized.

#### 2.4.2. Model 2: The absolute agreement formula ICC(A,1)

The equation system Eq (10) is over-determined, having five equations but only three unknowns (*σ*_*r*_, *σ*_*c*_ and *σ*_*v*_); two of the equations can be derived from the others. Solving Eq (10) for the *σ*:s we obtain (for example) the expressions

$$\begin{array}{l} \sigma_{r}{}^{2}\approx \frac{MSBS-MSE}{k}\\ \sigma_{c}{}^{2}\approx \frac{MSBM-MSE}{n}\\ \sigma_{v}{}^{2}\approx MSE \end{array}$$ (11)

Eq (11) provides us with a statistical estimate of the variances *σ*_*r*_^2^, *σ*_*c*_^2^ and *σ*_*v*_^2^. Using Eq (11) in Eq (9) we obtain

$$\begin{array}{l} \rho_{2A}=\frac{\sigma_{r}{}^{2}}{\sigma_{r}{}^{2}+\sigma_{c}{}^{2}+\sigma_{v}{}^{2}}\\ \approx \frac{MSBS-MSE}{MSBS+(k-1)\cdot MSE+(k/n)\cdot (MSBM-MSE)}\equiv ICC(A,1) \end{array}$$ (12)

i.e., the well-known sample ICC formula called ICC(A,1) [6], which is thus a statistical estimate of the population intraclass coefficient *ρ*_2A_. The formula ICC(A,1) gives an estimate of the reliability of the method if an absolute agreement between different measurements is desired [6]. Unlike the ICC(1) formula, it takes account also of the additional variance due to the randomly varying bias terms. We note that in the limit of vanishingly small bias terms, i.e. when *σ*_*c*_^2^ → 0, *MSBM*, *MSWS* and *MSE* tend to be equal since all three are then estimates of the same variance *σ*_*v*_^2^. Model 2 then reduces to Model 1 and the formula ICC(A,1) will tend to give the same result as the formula ICC(1).

#### 2.4.3 Model 2: The consistency formula ICC(C,1)

There are situations where a bias in the measurements, represented by the term *c*_*j*_, might be considered as acceptable. If a consistent ranking order of the subjects and a knowledge of the differences between them in each measurement is regarded as sufficient, we may choose to modify the expression Eq (9). Thus, a new population intraclass correlation coefficient is introduced, defined by [5]

$$\rho_{2C}=\frac{\sigma_{r}{}^{2}}{\sigma_{r}{}^{2}+\sigma_{v}{}^{2}}$$ (13)

The only difference from Eq (9) is that the variance *σ*_c_^2^ due to the bias in the measurements has been omitted. One may note the similarity to Model 1 (see Fig 1 or Eq (2)). The coefficient defined by Eq (13) is usually said to measure the consistency among the measurements [6], i.e. the extent to which the subjects maintain their ranking order and internal differences. It should be kept in mind that Model 2, given by Eq (8), is still assumed. Thus, even though the same model is used and the same measured data matrix is analyzed, the question asked (consistency rather than agreement) is different. Since one term in the denominator is missing, compared to Eq (9), we expect a higher ICC with Eq (13). This is reasonable from the intutitive point of view that if we are satisfied with just consistency, rather than absolute agreement, then the method might also be regarded as more satisfactory, i.e reliable, although in a restricted sense. It has been pointed out that Eq (13) is not properly an intra-class correlation coefficient, since the variance in the denominator is not the total one [26]. Still, it has been argued that it may be useful as a measure of consistency [5].

In any case, using the variance estimates Eq (11) in Eq (13) we get

$$\rho_{2C}=\frac{\sigma_{r}{}^{2}}{\sigma_{r}{}^{2}+\sigma_{v}{}^{2}}\approx \frac{MSBS-MSE}{MSBS+(k-1)\cdot MSE}\equiv ICC(C,1)$$ (14)

which is the standard formula for estimating consistency ICC, assuming Model 2 [6]. One advantage with this formula (and with ICC(A,1)) is that, unlike ICC(1), it is valid whether or not bias is present. We may note that if Model 1 is valid, then *MSWS* in the ICC(1) formula Eq (6) may be replaced by *MSE*, since *MSWS* and *MSE* are then equally valid estimates of *σ*_*v*_^2^ (Fig 1). We then obtain, of course, the ICC(C,1) formula.

#### 2.4.4. The lack of meaning of ICC(1) in the presence of bias

Suppose that a non-negligible bias is present, i.e. that Model 2 is appropriate. We may of course still calculate ICC(1) from an experimental matrix such as in Table 2, but what is the meaning of this ICC(1) value?

To see this, we apply Eq (10), i.e. the EMS relations in the presence of bias, to the ICC(1) formula Eq (6). This gives

$$\begin{array}{l} ICC(1)\equiv \frac{MSBS-MSWS}{MSBS+(k-1)\cdot MSWS}\approx \\ \frac{k\cdot \sigma_{r}{}^{2}+\sigma_{v}{}^{2}-(\sigma_{c}{}^{2}+\sigma_{v}{}^{2})}{k\cdot \sigma_{r}{}^{2}+\sigma_{v}{}^{2}+(k-1)\cdot (\sigma_{c}{}^{2}+\sigma_{v}{}^{2})}=\\ \frac{k\cdot \sigma_{r}{}^{2}-\sigma_{c}{}^{2}}{k\cdot (\sigma_{r}{}^{2}+\sigma_{c}{}^{2}+\sigma_{v}{}^{2})-\sigma_{c}{}^{2}}=\frac{\sigma_{r}{}^{2}-\sigma_{c}{}^{2}/k}{(\sigma_{r}{}^{2}+\sigma_{c}{}^{2}+\sigma_{v}{}^{2})-\sigma_{c}{}^{2}/k} \end{array}$$ (15)

Due to the presence of the two terms *σ*_*c*_^2^/*k* this is not equal to *ρ*_*2A*_ as defined by Eq (9), nor is it equal to *ρ*_1_ as defined by Eq (2). Thus, Eq (15) shows that the formula ICC(1), applied in the presence of bias, is not a correct estimate of any of the two population intraclass coefficients. In the presence of non-negligible bias, the ICC(1) formula should therefore not be used.

#### 2.4.5. The choice between Model 1 and Model 2

In a reliability study the method to be tested is normally designed with the intent that biases should not be present or at least negligible. However, the purpose of a reliability study is partly to check whether this is true. In practice the experimentalist can never be certain that bias effects are entirely absent.

When biases are absent, i.e. when Model 1 is valid, the ICC(A,1), ICC(C,1) and ICC(1) formulas are all estimates of the same population ICC, namely *ρ*_1_. We may therefore expect them to give at least nearly the same ICC value. Indeed, simulations (to be described below) indicate that the three ICC values will be approximately equal whenever Model 1 is valid. The simulations also indicate that if the ICC value is high (say, about 0.8 or higher), then the three ICC values will in fact be closely equal to each other.

On the other hand, when bias effects are present (Model 2) we may expect ICC(C,1) to be somewhat larger than ICC(A,1) (for the simple reason that *σ*_*c*_^2^ has been omitted from the denominator in the population ICC formula). Therefore, an ICC(C,1) value that is nearly equal to ICC(A,1) may be regarded as a qualitative sign that bias effects should be small or absent, while an ICC(C,1) value substantially larger than ICC(A,1) suggests the presence of non-negligible bias.

This is a convenient method of qualitative inspection. To confirm the presence of bias, a well-known statistical method is to use the *F*-value

$$F=\frac{MSBM}{MSE}$$ (16)

The average expected *F*-value is (see Fig 1)

$$F=\frac{MSBM}{MSE}\approx \frac{n\cdot \sigma_{c}{}^{2}+\sigma_{v}{}^{2}}{\sigma_{v}{}^{2}}=1+n\cdot {\left(\frac{\sigma_{c}}{\sigma_{v}}\right)}^{2}$$ (17)

In the presence of non-negligible bias (i.e., if *σ*_*c*_ is comparable to or larger than *σ*_*v*_) the *F*-value is thus expected to be larger than unity, possibly much larger (if *n* is large). If the probability under the null hypothesis (absence of bias) of finding an *F* value as high as the one calculated from the experimental matrix is very small, we may reject the null hypothesis, i.e. conclude that bias effects are probably present.

As seen from Eq (17), the sensitivity of this test depends on the strength of the biases and also, strongly, on the number of subjects *n*. If the biases are weak, i.e. *σ*_*c*_ << *σ*_*v*_, or if *n* is small, then the expected *F* value will be low and the F-test may fail to "detect" the presence of bias. On the other hand it should be noted that with a large *n*, the F-test might be significant even though biases are small. To clarify this in a practical case, the magnitude of the bias, i.e. the standard deviation *σ*_*c*_ (as compared to *σ*_*r*_ and *σ*_*v*_) can be estimated by means of Eq (11).

#### 2.4.6. Further comments on Model 2

McGraw and Wong [6] introduce a modified version of Model 2, called Case 2A, where the interaction term *rc*_*ij*_ is absent from Eq (7). With our description this makes no difference–the interaction term is included in the noise term–and the model is still equivalent to Eq (8). It thus leads again to the same formulas ICC(A,1) and ICC(C,1).

It may also be worthwhile to note that the sample ICC formulas can be written differently. For example, using the relations in S1 Appendix one can easily show that

$$ICC(A,1)=\frac{MSWM-MSE}{MSWM-MSE+MSWS}$$ (18)

This is not an independent estimate of *ρ*_*2A*_; Eq (18) is mathematically equivalent to the standard expression for ICC(A,1) shown in Eq (12). Thus, for a given matrix, it will give precisely the same value.

### 2.5. Model 3, leading to formula ICC(A,1) and formula ICC(C,1)

#### 2.5.1. Fixed biases

Model 3 is, as mentioned, described as a two-way mixed model [6], meaning that a random sampling of subjects is again assumed, while the biases are assumed to be fixed. We will introduce a theoretical modification that makes our Model 3 slightly different from the ICC(3,1) case of Shrout and Fleiss [5] as well as the Case 3 model described by McGraw and Wong [6]. However, we still obtain precisely the same ICC formulas. We should add that we will finally argue (in Section 6) that in a reliability study there is actually no difference between Model 2 and Model 3.

Formally, the conventional Model 3 looks at first sight just like Eq (7), i.e.

$$x_{ij}=\mu +r_{i}+c_{j}+rc_{ij}+e_{ij}$$ (19)

The *μ*, *r*_*i*_ and *e*_*ij*_ are defined as before, and *r*_*i*_ and *e*_*ij*_ are sampled as before from normal distributions with mean values = 0 and standard deviations *σ*_*r*_ and *σ*_*e*_. However, the bias terms *c*_*j*_ and the interaction terms *rc*_*ij*_ are different.

In Model 3, the terms *c*_*j*_ in Eq (19) are assumed to be fixed rather than sampled from a normal distribution. The meaning of this is perhaps confusing. As mentioned, it will be argued that in the analysis of a reliability study there is no difference between Model 2 and Model 3, i.e. there is no difference between fixed and random biases. This may be intuitively understood from the fact when a matrix such as indicated in Table 2 is generated using Eq (19), it does not matter for the subsequent calculation of an ICC value whether the constants *c*_*j*_ (*j* = 1, 2, … *k*) are randomly sampled from a normal distribution or chosen in some other way.

It may however be meaningful to speak about fixed bias terms *c*_*j*_ if they remain the same when the measurements are continued with new subjects. An example, given by Shrout and Fleiss [5] and commonly referred to in the literature, is the situation where raters are judging subjects and where "the selected raters are the only raters of interest" [5]. In practice, this should mean that the same raters (*j* = 1,2, …, *k*) are used in subsequent measurements.

Turning to the value of the interaction term *rc*_*ij*_, it is assumed to be sampled from a normal distribution with mean = 0 and standard deviation *σ*_*rc*_. This would seem to be equivalent to Model 2. However, in the conventional approach [5,6] to Model 3 there is a restriction on *c*_*j*_ as well as *rc*_*ij*_, which is discussed in the next section.

#### 2.5.2 Model 3, conventional approach: fixed biases restricted to sum = zero

In the Case 3 model described by Shrout and Fleiss [5] and McGraw and Wong [6], the *c*_*j*_ are, unfortunately without any explicit motivation or any reference to literature, subjected to the restriction

$$\sum_{j}c_{j}=0$$ (20)

Thus, if *k* = 3, say, and *c*_1_ = 1, *c*_2_ = 1, then, necessarily *c*_3_ = -2. Likewise, the *rc*_*ij*_ are assumed to be sampled from a normal distribution with a standard deviation *σ*_*rc*_, however with a similar restriction

$$\sum_{j}rc_{ij}=0$$ (21)

Eq (21) means that for each subject (*i*) the interaction terms, although varying from subject to subject, are required to add up to zero. This leads to a mathematical complication referred to as anti-correlation [5]. Bartko [4], however, apparently introduces Model 3 without these restrictions. We feel that the restrictions Eq (20) and Eq (21) should be critically discussed.

First, we note that their effect is to guarantee that the average value of *x*_*ij*_ obtained with a finite number of measurements (e.g., *k* = 3) but a very large (*n* → ∞) number of subjects will, according to Eq (19), be equal to the population mean value *μ*. (The mean values of *r*_*i*_ and *e*_*ij*_, each being sampled from a normal distribution with mean value = 0, will tend to zero). Model 1 and Model 2 do have this property, as may be seen from Eq (1) and Eq (8); in the case of Model 2 this is so because the *c*_*j*_ values are sampled from a normal distribution with the mean value = 0.

However, we find that the restrictions Eq (20) and Eq (21) are artificial from an experimental point of view. If, for example, a limited number *k* of raters are selected (randomly or not) from a population of raters, their *c*_*j*_ values will in general *not* add up to zero. (This is obvious when performing a simulation based on Model 2.) Thus, in the presence of fixed biases, the average value of *x*_*ij*_ should differ systematically from the population average *μ* of the true scores. The departure is due to the biased method of measurement and cannot be eliminated by increasing the number of subjects. The same argument may be applied to the *rc*_*ij*_ term; in a single matrix with a finite number of subjects and measurements, the interaction terms *rc*_*ij*_ should, just like *e*_*ij*_, in general *not* add up to zero; as is again obvious when looking back at Model 2.

Eq (20) may be questioned also from a mathematical point of view. Suppose that the bias terms *c*_*j*_ are modified by adding to each of them the same arbitrary constant *C*; the new bias terms are thus *c*_*j*_ *+ C*. This will make the matrix elements *x*_*ij*_ different (*x*_*ij*_ → *x*_*ij*_ + *C*). However, using the definitions and relations in S1 Appendix it can be shown that the sums of squares (*SST*, *SSBS*, etc.) are not changed by this modification. It follows that also the mean squares and thus the estimated variances and sample ICC values calculated from the matrix will be precisely the same as before.

Now, let *c*_*j*_ be arbitrary bias terms. In general, they may not satisfy Eq (20). We obtain bias terms satisfying Eq (20) by adding to each *c*_*j*_ a common constant, namely $-\bar{c}$, where $\bar{c}$ is their average value. However, it is clear from the above observation that this will not change the mean squares or the ICC values calculated from the matrix.

Conversely, assume that the bias terms originally do satisfy Eq (20). Suppose we modify them by adding to each of them the same but arbitrary constant C ≠ 0. Then Eq (20) will no longer be satisfied, but the mean squares and ICC values will remain precisely the same.

We conclude that the restriction Eq (20) may be omitted without changing the results of Model 3 as regards mean squares and intraclass correlations. What matters is the distribution of the *c*_*j*_ values relative to their average value $\bar{c}$.

#### 2.5.3. Model 3, present approach: No restriction

In the presently discussed version of the two-way mixed model (Model 3), we will therefore not apply the restrictions Eq (20) and Eq (21). This means a considerable simplification of the theory. It will still lead to the same ICC formulas as those described for Model 3 by McGraw and Wong [6], i.e., to ICC(A,1) and ICC(C,1).

In the absence of the restriction Eq (21), and thus in the absence of anti-correlation, we may again replace *rc*_*ij*_ and *e*_*ij*_ in Eq (19) by a single “noise” term *v*_*ij*_ sampled from a normal distribution with standard deviation *σ*_*v*_. Thus, Model 3 becomes simply

$$x_{ij}=\mu +r_{i}+c_{j}+v_{ij}$$ (22)

where, however, the *c*_*j*_ now are fixed. The absolute agreement population ICC with Model 3 is therefore similar to Eq (9), i.e. to that obtained with Model 2, except that (according to the conventional approach) the Model 2 bias term variance *σ*_*c*_^*2*^ is replaced (see S2 Appendix) by

$$\theta_{c}{}^{2}=\frac{\sum_{j}{\left(c_{j}-\bar{c}\right)}^{2}}{\left(k-1\right)}$$ (23)

i.e. the variance of the fixed bias terms *c*_*j*_ about their average $\bar{c}$. The absolute agreement population ICC with Model 3 is thus

$$\rho_{3A}=\frac{\sigma_{r}{}^{2}}{\sigma_{r}{}^{2}+\theta_{c}{}^{2}+\sigma_{v}{}^{2}}$$ (24)

Eq (24) is identical to the population ICC named "Case 3A, absolute agreement" by McGraw and Wong [6]. From S2 Appendix we find the EMS relations for Model 3 to be

$$\begin{array}{l} MSBS\approx k\cdot \sigma_{r}{}^{2}+\sigma_{v}{}^{2}\\ MSBM\approx n\theta_{c}{}^{2}+\sigma_{v}{}^{2}\\ MSWS\approx \theta_{c}{}^{2}+\sigma_{v}{}^{2}\\ MSWM\approx \sigma_{r}{}^{2}+\sigma_{v}{}^{2}\\ MSE\approx \sigma_{v}{}^{2} \end{array}$$ (25)

Solving Eq (25) for *σ*_*r*_^2^, *σ*_*c*_^2^ and *σ*_*v*_^2^ we get the variance estimates

$$\begin{array}{l} \sigma_{r}{}^{2}\approx \frac{MSBS-MSE}{k}\\ \theta_{c}{}^{2}\approx \frac{MSBM-MSE}{n}\\ \sigma_{v}{}^{2}\approx MSE \end{array}$$ (26)

Using Eq (26) in Eq (24) we obtain precisely the same ICC(A,1) formula as the one we obtained with Model 2, i.e., Eq (12). This result was also reached by McGraw and Wong [6]. It is not surprising, in view of the fact that in going from Model 2 to Model 3, we have replaced *σ*_*c*_^*2*^ by *θ*_*c*_^*2*^ in the EMS relations Eq (25) as well as in the definition of the absolute agreement ICC, Eq (24).

We now turn to the consistency version of the population intraclass correlation coefficient, which with Model 3 is defined in the same way as for Model 2, i.e. as

$$\rho_{3C}=\frac{\sigma_{r}{}^{2}}{\sigma_{r}{}^{2}+\sigma_{v}{}^{2}}$$ (27)

Remembering that *σ*_*v*_^2^ = *σ*_*e*_^2^ + *σ*_*rc*_^2^, Eq (27) is identical to the population ICC referred to as "Case 3A, consistency" by McGraw and Wong [6]. In fact, Eq (27) is identical to Eq (13), and using Eq (25) it gives again the ICC(C,1) formula of Eq (14), also in agreement with McGraw and Wong [6]. This result is also reached by Bartko [4], with the added assumption that the interaction term is negligibly small. This assumption has not been used here.

**Fig 2:**
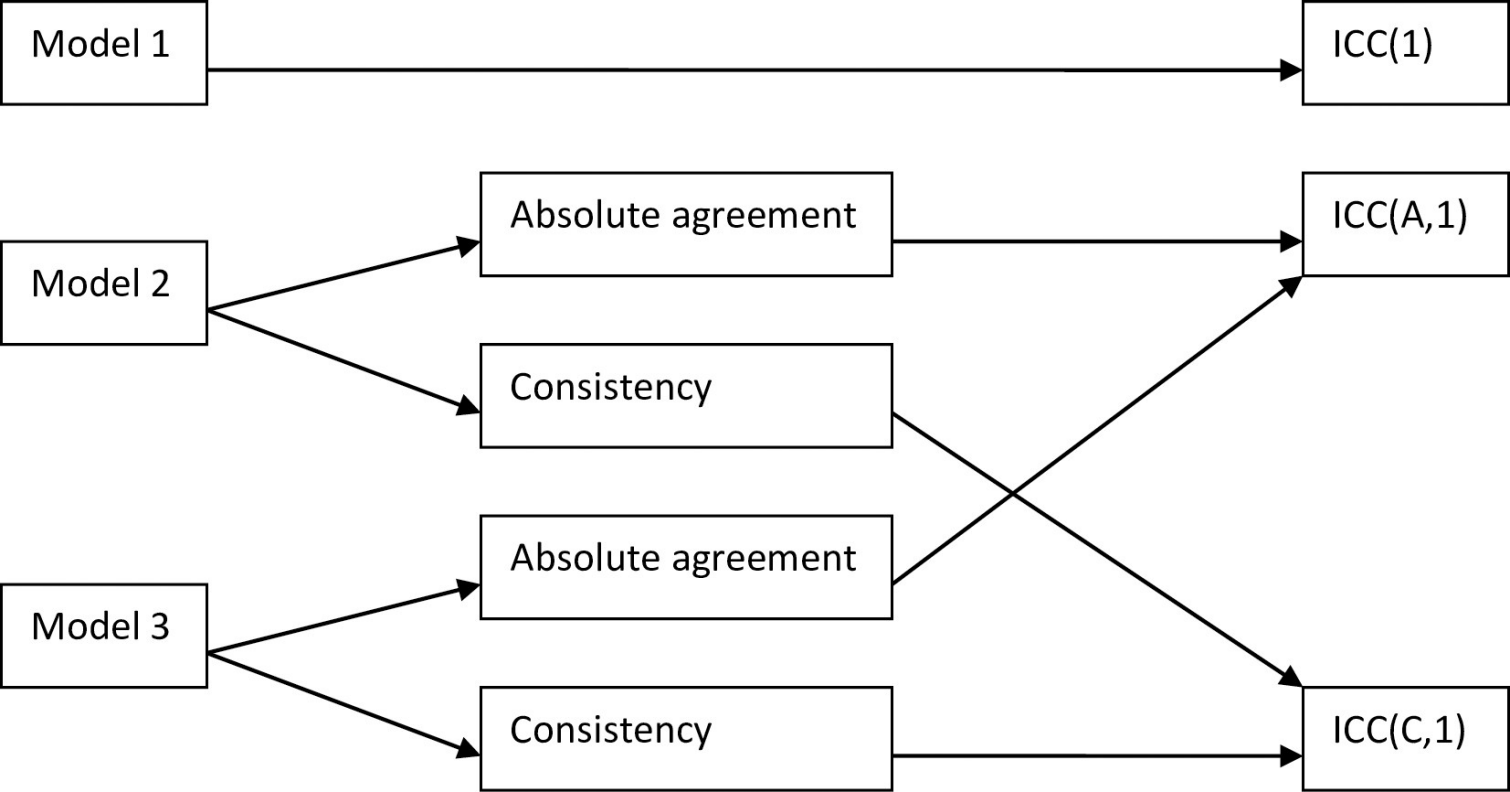
Relation between ICC models and ICC formulas. Three statistical models used in the intraclass correlation theory are indicated: Model 1 (one-way model); Model 2 (two-way random model); and Model 3 (two-way mixed model). The figure shows the relation between these models and the three well-known sample ICC formulas, i.e. ICC(1), ICC(A,1) and ICC(C,1).

Thus, if a choice is to be made between the two-way random and the two-way mixed models one should be aware that both models lead to precisely the same sample ICC formulas. The same absolute agreement ICC and consistency ICC values will be obtained for a given data matrix, regardless of which model is regarded as appropriate. One may therefore reasonably wonder whether there really is any difference between Model 2 and Model 3. As mentioned, we will return to this question in Section 6. We will also demonstrate fixed bias results by Monte Carlo simulation in Section 4. A flow diagram summarizing the connection between models and the resulting formulas is shown in Fig 2.

One choice which definitely does make a difference in the value of the ICC (unless bias are very small or absent) is the choice between the absolute agreement formula ICC(A,1) and the consistency formula ICC(C,1) (Fig 2). That choice may seem to depend only on whether absolute agreement is desired or if consistency is sufficient. However, there is one further aspect that should be noted.

If the biases, i.e. the numbers *c*_*j*_, are fixed in continued measurements, then a reasonable experimentalist approach should be either to try to correct the measured values *x*_*ij*_ for these biases, or to eliminate (remove) the sources of bias. Let the corrected *x*_*ij*_ value be called *y*_*ij*_. Then, according to Eq (22), the corrected values are given by

$$y_{ij}=x_{ij}-c_{j}=\mu +r_{i}+v_{ij}$$ (28)

But from this equation it is clearly seen that the corrected values *y*_*ij*_ may in fact be generated by Model 1, i.e. without bias. The appropriate population intraclass correlation coefficient for the corrected values should therefore be given by Eq (27), i.e. that coefficient which is estimated by ICC(C,1). Consequently, one may regard ICC(C,1) as an estimate of the population intraclass correlation coefficient that would be obtained if the bias terms could be eliminated or corrected for. This, which applies to Model 2 as well as Model 3, seems to be a somewhat stronger statement than saying merely that ICC(C,1) is a measure of consistency. It will be clearly demonstrated also by the simulations discussed below. In fact, it was proposed by Alexander already in 1947 [17].

### 2.6. Models specified from experimental design

Much discussion has been devoted to the question of which model (Model 1, 2 or 3) should be specified, i.e. correctly associated with a given experimental design [2,4,5,6,19,25,26,27]. We wish to show that a simple argument based on the algebraic formulas that define the models may be helpful. Neverthless, we also wish to point out that it will later be shown that such a specification of a model should be considered to be only preliminary and may have to be reconsidered in the light of experimental data.

In this discussion it will be convenient to differ between biases associated with raters and biases associated with the measurement procedure (for example, effects of learning or fatigue in a series of measurements on the same subject). We will indicate rater biases with a single prime, for example *c'*_*j*_, while procedure biases are indicated by a double prime, for example *c''*_*j*_. With a linear model such as assumed in Eq (1) and Eq (8), the total bias will be the sum of the two kinds of biases, i.e. *c*_*j*_ *= c'*_*j*_ *+ c''*_*j*_.

***Example 1*: *one rater each column*.** Each of *n* subjects is judged (measured) once by each of *k* raters. (This design may be used e.g. in an inter-rater reliability study.) There might be non-zero rater biases *c'*_*j*_ as well as procedure biases *c''*_*j*_. A measured value should be the sum of all terms, i.e.

$$x_{ij}=\mu +r_{i}+{c^{\prime }}_{j}+{c^{\prime \prime }}_{j}+v_{ij}=\mu +r_{i}+c_{j}+v_{ij}$$ (29)

We can see that Eq (29) has the form of Eq (8). This means that Model 2 or 3 are expected to be appropriate, depending on whether *c*_*j*_ is regarded as random or fixed. We note that with this design, rater biases cannot be separated from procedure biases. If both biases are absent (*c'*_*j*_ = *c''*_*j*_ = 0) we get, of course, Eq (1); then Model 1 is appropriate.

***Example 2*: *one single rater*.** Each of *n* subjects is judged (measured) *k* times. There is only one rater, with a single bias we may call c'; this rater performs all the measurements. This design might be used e.g. in an intra-rater reliability study; it is also common in medical reliability studies where the identity of the rater is not regarded as important. We first assume that there are no procedure biases, i.e. *c*_*j*_*''* = 0. Then

$$x_{ij}=\mu +r_{i}+c^{\prime }+v_{ij}=(\mu +c^{\prime })+r_{i}+v_{ij}=\mu^{\prime }+r_{i}+v_{ij}$$ (30)

Only the apparent population mean value is affected, being shifted from *μ* to *μ'* = *μ + c'*. Eq (30) has the form of Eq (1), i.e. Model 1 is expected to be appropriate. On the other hand, if there are procedure biases, i.e. if not all the *c*_*j*_*''* are zero, then a term *c*_*j*_*''* should be added to Eq (30). We then recognize Eq (8), i.e. Model 2 or 3 should be appropriate.

***Example 3*: *one rater for each subject*.** Each subject is measured *k* times by the same rater, but different subjects have different raters (for example from different clinics). Thus, there are *n* raters, and the rater biases are indexed as *c'*_*i*_, *i* = 1, 2, … *n* according to which subject they are measuring. Assume there are no procedure biases. This gives

$$x_{ij}=\mu +r_{i}+{c^{\prime }}_{i}+v_{ij}=\mu +{r^{\prime }}_{i}+v_{ij}$$ (31)

where *r'*_*i*_ *= r*_*i*_ *+ c*_*i*_. We recognize Eq (1), so Model 1 is expected to be appropriate, but we should observe from Eq (31) that what is estimated is a population ICC given by

$$\rho =\frac{{\sigma^{\prime }}_{r}{}^{2}}{{\sigma^{\prime }}_{r}{}^{2}+\sigma_{v}{}^{2}}$$ (32)

where *σ'*_*r*_^*2*^ *= σ*_*r*_^*2*^ *+ σ*_*c*_^*2*^, i.e. the sum of the variance of the subject's true scores and the variance of the raters; these two variances cannot be separated with such an experimental design.

Now suppose that procedure biases *c''*_*j*_ are present. Adding this term to Eq (31) shows that Model 2 or 3 are then expected to be appropriate.

***Example 4*: *A different rater for each subject and each measurement*.** This should be a very unusual design, but it may be shown to illustrate the principle of the argument. Each of the *n* × *k* measurements is made by a different rater with a bias *c'*_*ij*_ sampled from a normal distribution with variance *σ*_*c*_^*2*^; thus, there are *n* × *k* raters. Assume that there are no procedure biases. This gives

$$x_{ij}=\mu +r_{i}+{c^{\prime }}_{ij}+v_{ij}=\mu +r_{i}+{v^{\prime }}_{ij}$$ (33)

where *v'*_*ij*_ *= c'*_*ij*_ *+ v*_*ij*_ is sampled from a normal distribution with variance *σ'*_*v*_^*2*^ *= σ*_*c*_^*2*^ *+ σ*_*v*_^*2*^. Eq (33) has the form of Eq (1), which shows that Model 1 is expected to be appropriate, but it should be observed that the noise term includes the random variation of rater bias. The random variation of rater biases cannot be separated from noise.

### 2.7. What ICC value is high enough?

Qualitative interpretations of ICC values are often recommended, for example (under certain restrictions [2]) “poor” (ICC < 0.5), "moderate" (0.5–0.75), “good” (0.75–0.9) and "excellent" (ICC > 0.9). However, there are also quantitative ways of considering an ICC value obtained from a reliability study. We take Model 1 as an example. The population ICC given by Eq (2) may be written.

$$\rho_{1}=\frac{1}{1+{\left(\sigma_{v}/\sigma_{r}\right)}^{2}}$$ (34)

Suppose that it is required that the typical error or uncertainty in the method of measurement should be less than 10% of the spread among the true scores, i.e., *σ*_*v*_ < 0.1*σ*_*r*_. (One example could be the measurement of body temperature, where a typical range of interest might be a few degrees, while the measurement of the temperature may have an uncertainty of about 0.1°C.) Inserting *σ*_*v*_ < 0.1*σ*_*r*_ into Eq (34) we find that the population ICC should then be about 1/(1 + 0.01) ≈ 0.99 or larger. The required ICC value is thus very close to unity. However, it may be questioned if an ICC value that close to unity is informative; rather, one should consider the ratio *σ*_*v*_ /*σ*_*r*_.

Suppose instead that we assume that *ρ*_1_ > 0.8 is high enough. Using this in the above equation gives *σ*_*v*_/*σ*_*r*_ < 0.5. This means that the error *σ*_*v*_ might be as much as 50% of the spread *σ*_*r*_ among the true scores. Such a large error might be acceptable in some contexts but not in others; consequently, we may have to reconsider our assumption. A higher ICC value might be required.

Considerations such as these might be of assistance when judging if an ICC value is high enough in a given clinical context.

## 3. Method Part II: Simulation

### 3.1. Simulation of ICC probability distributions from Model 1

Monte Carlo simulation is well-known in statistical research [28–31]. Still, a brief comment on the difference between for example a clinical reliability test and the present simulation is motivated.

In a typical clinical or research reliability test, the researcher starts by planning an experiment according to a specific design. Subjects willing to take part in the study have been found, inclusion and exclusion criteria have been applied and ethical permissions have been secured. Finally, measurements have been made, resulting in an experimental data matrix of size *n* × *k* (Table 2) from which the researcher, using ANOVA and a specified statistical model corresponding to the design, may derive a standard error of measurement (*σ*_*v*_), an ICC value and a confidence interval for the population ICC.

In the simulation we do essentially the reverse. We start from a specified statistical model (Model 1, 2 or 3). Using this model, we let a computer simulate (create) "experimental" data which then are analyzed. For the simplest model, Model 1, we begin by choosing explicit numerical values for the population mean value *μ*, the population standard deviations *σ*_*r*_ and *σ*_*v*_, the number of subjects *n* and the number of measurements *k*. In order to study essentially all possible results of this model, a large number *N* of "experimental" data matrices are simulated, each of size *n* × *k*. In the present simulations *N* = 10000 for each case of interest; this provides sufficient statistical accuracy for our purposes.

The simulation of each "experimental" data matrix is for Model 1 performed in accordance with Eq (1). Thus, each matrix element *x*_*ij*_ is explicitly calculated to be the sum of a population mean value *μ*, a signal term *r*_*i*_ and a noise term *v*_*ij*_. The values *r*_*i*_ are randomly sampled from a normal distribution with mean value zero and the chosen standard deviation *σ*_*r*_, while the values *v*_*ij*_ are randomly sampled from a normal distribution with mean value zero and the chosen standard deviation *σ*_*v*_. (The element of randomness in the simulation is the reason for the traditional name “Monte Carlo simulation”.) For transparency and completeness, the method presently used for this random sampling is briefly indicated in Appendix 3 (S3 Appendix).

Each simulated "experimental" matrix will be a little different because of the random nature of the *r*_*i*_ and the *v*_*ij*_ numbers. When finished, it is (within the simulation program) subjected to ANOVA. An ICC(1) value and an *F* value are calculated and stored; for comparison, ICC(A,1) and ICC(C,1) values are calculated and stored as well. Thus, in one single simulation with *N* = 10000 we get, for example, a set of 10000 simulated ICC(1) values. This set may be regarded as a probability distribution of the ICC(1) values generated by the specified model. From the ICC(1) distribution the average ICC(1) value is calculated and its 95% central range limits (see below) are estimated numerically. Apart from a minute for manually feeding the input data (*μ*, *σ*_*r*_, *σ*_*v*_, *n*, *k*, and *N* in the case of Model 1), the simulation and analysis of *N* = 10000 “experiments” takes typically one or a few seconds on an ordinary personal computer.

The advantage of such simulations is that they may help us to understand how the ICC “works”, we may get a survey of what to expect in an experiment, and we may better understand how to make a proper choice of models, experimental designs and analysis.

### 3.2. Data obtained from a Model 1 ICC probability distribution

From the *N* = 10000 simulated mean squares, ICC values and F values various averages are calculated. For example, let *ICC*_*J*_ (1) be the sample ICC(1) value obtained from the *J*:th simulated matrix. The average ICC(1) value is then

$$<ICC(1)>=\frac{1}{N}\sum_{J=1}^{N}ICC_{J}(1)$$ (35)

The same notation (< … >) is used for other averages over the *N* simulated matrices. Thus, for example <*MSBS*> is, as mentioned, the average mean square between subjects. These averages are presented together with a standard deviation calculated in the usual manner. The standard deviations are only meant to give a rough idea of the spread of simulated data around the average value.

The confidence limits of the population ICC are of greater interest. In order to estimate these confidence limits, the 95% central range limits of the simulated ICC(1) distributions may be used. With a large number *N* of simulated ICC(1) values, 2.5% are expected to be above the upper central range limit, while 2.5% are expected to be below the lower central range limit. In the present simulations, a simple numerical method has been used to estimate these limits: with *N* = 10000, the upper 95% central range limit is estimated as being equal to the lowest simulated ICC(1) value among the 250 highest ones, while the lower limit is estimated as being equal to the highest ICC(1) value among the 250 lowest ones. The connection between these sample ICC central range limits and the population ICC confidence interval will be demonstrated in Section 4.

It is informative to study for example the average value <ICC(1)> and the central range limits as functions of the input data. For a systematic survey of ICC(1) we note again that the population intraclass coefficient for Model 1 may be written as in Eq (34), from which it is obvious that *ρ*_1_ depends only on the ratio *σ*_*v*_ /*σ*_*r*_. All cases of interest may therefore be simulated and studied by varying this ratio within a reasonable interval, for example 0 < (*σ*_*v*_ /*σ*_*r*_)^2^ < 2. (A value of (*σ*_*v*_ /*σ*_*r*_)^2^ larger than 2 is deemed not to be of interest since the expected average ICC(1) value would then be less than 0.33.)

It is also obvious from Eq (34) that, as regards the ICC value, the separate values of *μ*, σ_*r*_ and σ_*v*_ do not matter. We may therefore, without any loss of generality, choose fixed values for *μ* and σ_*r*_, while varying σ_*v*_, i.e. the ratio *σ*_*v*_ /*σ*_*r*_. For convenience we have therefore throughout all present simulations assumed *μ* = 100 and σ_*r*_ = 10. For example, a value of σ_*v*_ = 5 will then give (*σ*_*v*_ /*σ*_*r*_)^2^ = 0.25 and *ρ*_1_ = 0.8.

### 3.3. Methods for simulation of Model 2 and Model 3

The methods are similar to that used for Model 1. In Model 2 each matrix element is calculated by the formula Eq (8), i.e. as a sum of a population mean value *μ*, a signal term *r*_*i*_, a bias term *c*_*j*_ and a noise term *v*_*ij*_. The sampling of the *r*_*i*_ and *v*_*ij*_ terms are performed as for Model 1. The terms *c*_*j*_ are sampled from a normal distribution with standard deviation σ_*c*_ and mean value zero. Input data for the simulation are thus *N*, *n*, *k*, *μ* and the standard deviations σ_*r*_, σ_*c*_ and σ_*v*_. For each simulated matrix, mean squares (*MS*) and the ICC(A,1) and ICC(C,1) values are calculated. For comparison, we also calculate ICC(1), even though it is not valid when biases are present.

For a systematic survey of ICC(A,1) we may note that the population ICC for absolute agreement may be written

$$\rho_{2A}=\frac{1}{1+{\left(\sigma_{c}/\sigma_{r}\right)}^{2}+{\left(\sigma_{v}/\sigma_{r}\right)}^{2}}$$ (36)

This depends on two ratios, namely *σ*_*c*_ /*σ*_*r*_ and *σ*_*v*_ /*σ*_*r*_; for the survey, these ratios may be varied independently over suitable intervals.

In Model 3, each matrix element *x*_*ij*_ is calculated as a sum of a population mean value *μ*, a signal term *r*_*i*_, a fixed bias term *c*_*j*_ (i.e., equal for all the simulated matrices) and a noise term *v*_*ij*_. The signal term *r*_*i*_ and the noise term *v*_*ij*_ are sampled from their respective normal distributions. Input data for the simulation are thus *N*, *n*, *k*, *μ*, the standard deviations σ_*r*_ and σ_*v*_, and the *k* fixed (but arbitrary) *c*_*j*_ values. Again, we use *N* = 10 000, *μ* = 100 and *σ*_*r*_ = 10 throughout. There is an infinite number of ways of choosing the fixed *c*_*j*_ values. For our purposes it will be sufficient to study only two cases.

### 3.4. Fortran code

An interactive Fortran 77 code, SIMANOVA, was written to perform these simulations. It was repeatedly rewritten in order to perform the various tasks given to it. The source code of a rather general version is given in Appendix 4 (S4 Appendix). Output data for plotting are given in a form suitable e.g. for Excel. Note that two random seeds have to be given to the random generator, for example two four-digit integers.

## 4. Simulation studies

### 4.1. Simulation study of Model 1

**Fig 3:**
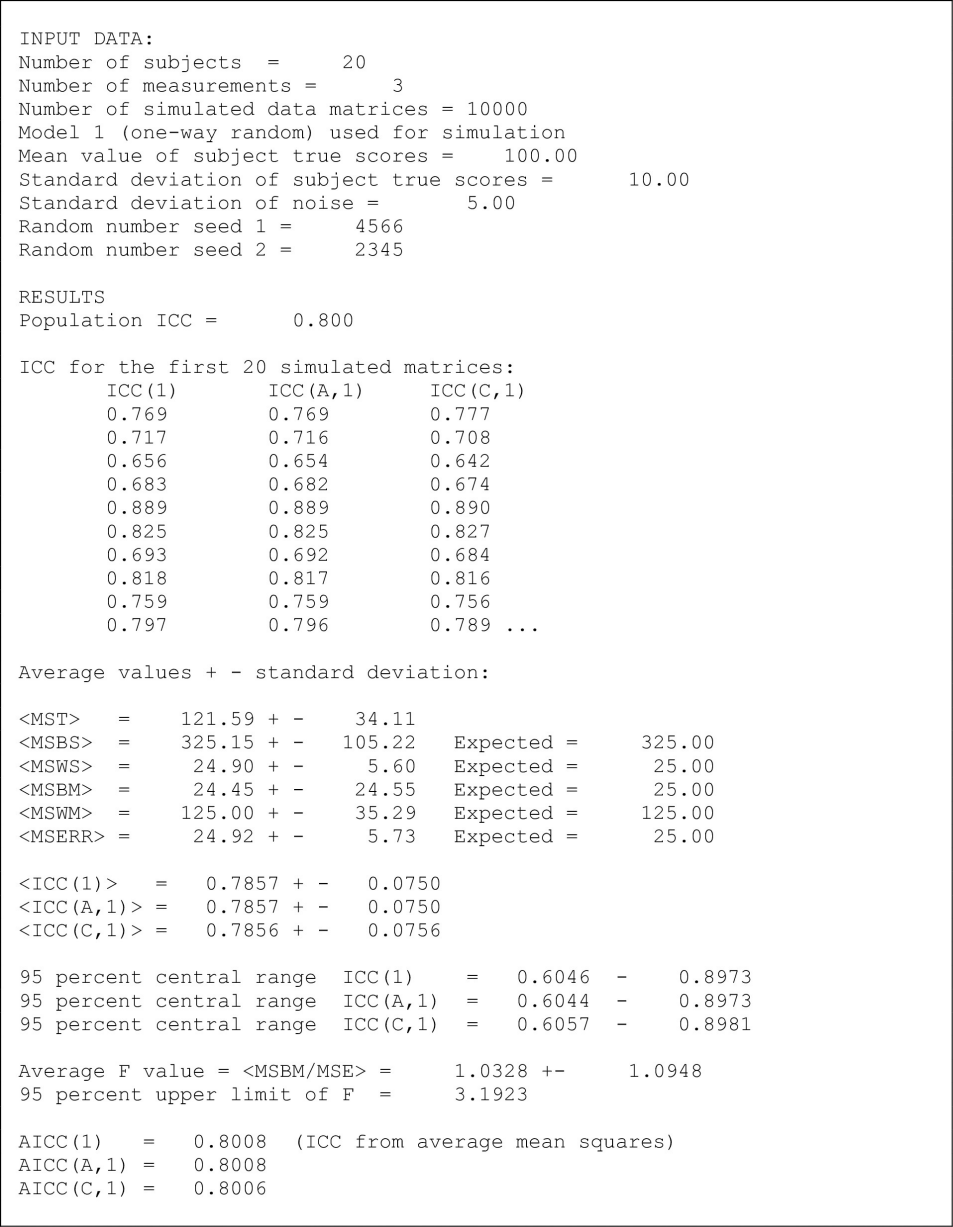
Part of the computer printout from a SIMANOVA run using Model 1. Corresponding ICC distributions are shown in Fig 4. See text for discussion.

**Fig 4:**
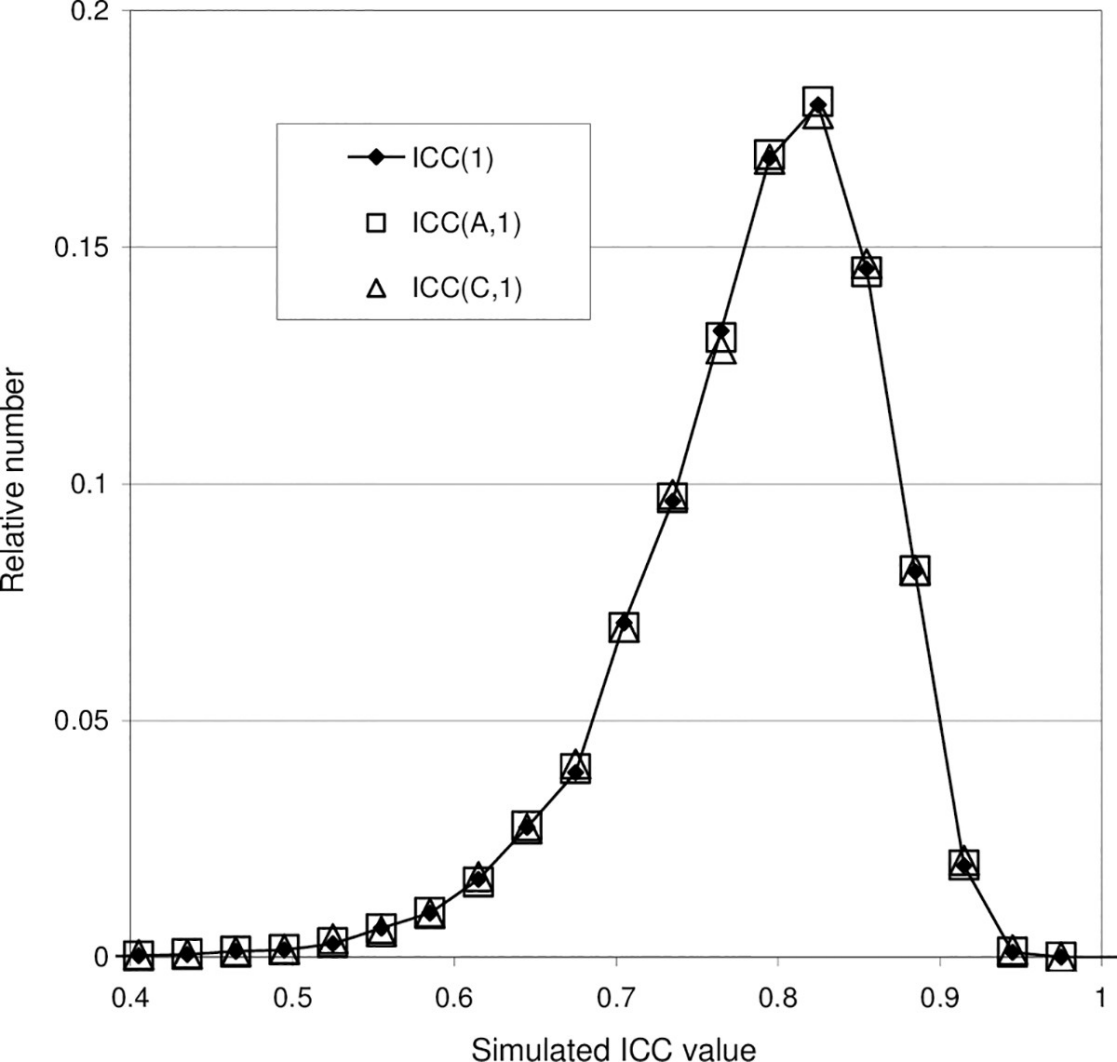
Probability distributions of ICC(1), ICC(A,1) and ICC(C,1) obtained with a simulation based on Model 1, i.e. in the absence of bias. Input data and results from this simulation are shown in Fig 3. With Model 1, the simulated distributions are seen to be identical apart from small differences due to finite statistics (finite *N*).

Fig 3 shows results (part of the print-out, somewhat edited) of one specific Monte Carlo simulation run using Model 1. Fig 3 (as well as Fig 4; see text below) may be reproduced by running SIMANOVA with the input data shown, including the random seeds. As seen from Fig 3, input data in this run were *n* = 20, *k* = 3, *N* = 10 000, *μ* = 100, *σ*_*r*_ = 10, and *σ*_*v*_ = 5, with the two random seeds 4566 and 2345. A different choice of random seeds will give statistically the same result, but not exactly the same numbers. As mentioned, the values *μ* = 100, *σ*_*r*_ = 10 and *N* = 10000 are used throughout all simulations.

From Eq (34) the population intraclass correlation coefficient is *ρ*_1_ = 1/(1 + 5^2^/10^2^) = 0.8. In a similar way, the expected average mean squares *MSBS*, *MSWS*, *MSBM*, *MSWM* and *MSE* are calculated using the EMS relations (see Fig 1) from the variances *σ*_*r*_ = 10, and *σ*_*v*_ = 5; their values are shown in Fig 3. The simulated average mean square values (< *MSBS* >, etc.) are in excellent agreement with the expected values, apart from very small statistical fluctuations due to the finite (albeit large) value of *N*.

For each of the *N* matrices, all three sample ICC values ICC(1), ICC(A,1) and ICC(C,1) are calculated. Not only do their average values <ICC(1)>, <ICC(A,1)> and <ICC(C,1)> agree very closely, as should be expected with Model 1, but the values tend in fact to agree closely also within each single simulated matrix. This is exemplified in Fig 3 by the result for the 10 first simulated matrices.

It may be noted that all three average ICC values are slightly different from the population ICC value = 0.8. This is due to a simple numerical effect. The average ICC(1), for example, is given by

$$<ICC(1)>=<\frac{MSBS-MSWS}{MSBS+(k-1)\cdot MSWS}>$$ (37)

In general, this is not precisely equal to the alternative expression

$$AICC(1)=\frac{<MSBS>-<MSWS>}{<MSBS>+(k-1)\cdot <MSWS>}$$ (38)

(i.e., it does matter whether the average is taken before the division or after.) Since the average simulated *MS* values in Eq (38) agree very well with the expected *MS* values, AICC(1) agrees better with the population ICC, *ρ*_1_, than does <ICC(1)>. However, <ICC(1)> is by definition the average ICC(1) value calculated from the ICC(1) distribution. Moreover, the difference between the values calculated by Eq (37) and Eq (38) is in practice small compared to the widths (95% central ranges) of the simulated ICC distributions. A similar argument applies to ICC(A,1) and ICC(C,1). The simulated 95% central ranges are shown in Fig 3 for all three calculated ICC versions.

For the same simulation run as shown in Fig 3, Fig 4 shows the simulated distributions of the values of ICC(1), ICC(A,1) and ICC(C,1). The distributions are presented as histograms, for graphical clearness with points (symbols) rather than staples. The relative number *N*_*i*_/*N*, where *N*_*i*_ is the number of simulated ICC values found in the histogram channel (interval) *i*, is plotted against the value of the ICC corresponding to the center of the channel. Thus, the simulated distributions may be regarded as estimates of the normalized probability distributions of the ICC values for the given values of the model parameters. It can be seen that the three distributions obtained with Model 1 are nearly identical, apart from very small statistical variations. The distribution maxima are positioned near the population ICC = 0.8.

**Fig 5:**
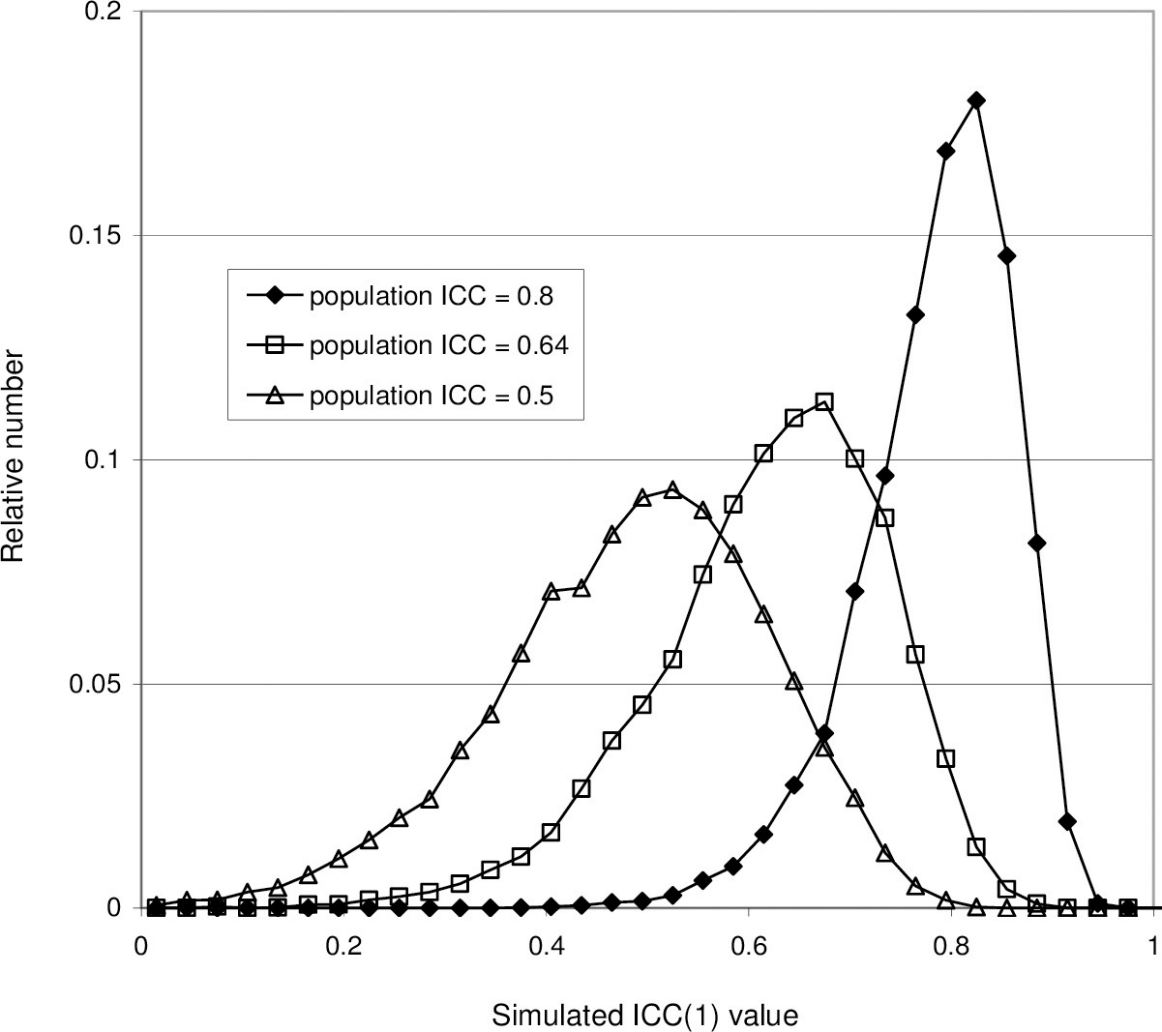
Probability distributions of ICC(1) values obtained with simulations using Model 1, showing the effect of increasing noise (error). In all three cases *n* = 20, *k* = 3 and *σ*_*r*_ = 10. The standard deviation of the noise term is increased from *σ*_*v*_ = 5 (giving population ICC *ρ*_1_ = 0.8) to *σ*_*v*_ = 7.5 (*ρ*_1_ = 0.64) and *σ*_*v*_ = 10 (*ρ*_1_ = 0.5).

The effect of increasing the noise (interaction + error), i.e. increasing the standard deviation *σ*_*v*_ while keeping everything else fixed, is shown in Fig 5. As follows from Eq (2), the population intraclass coefficient *ρ*_1_ then decreases. In accordance with this, the ICC(1) distribution shifts to a lower average value < ICC(1) > and is widened as well, increasing its 95% central range.

**Fig 6:**
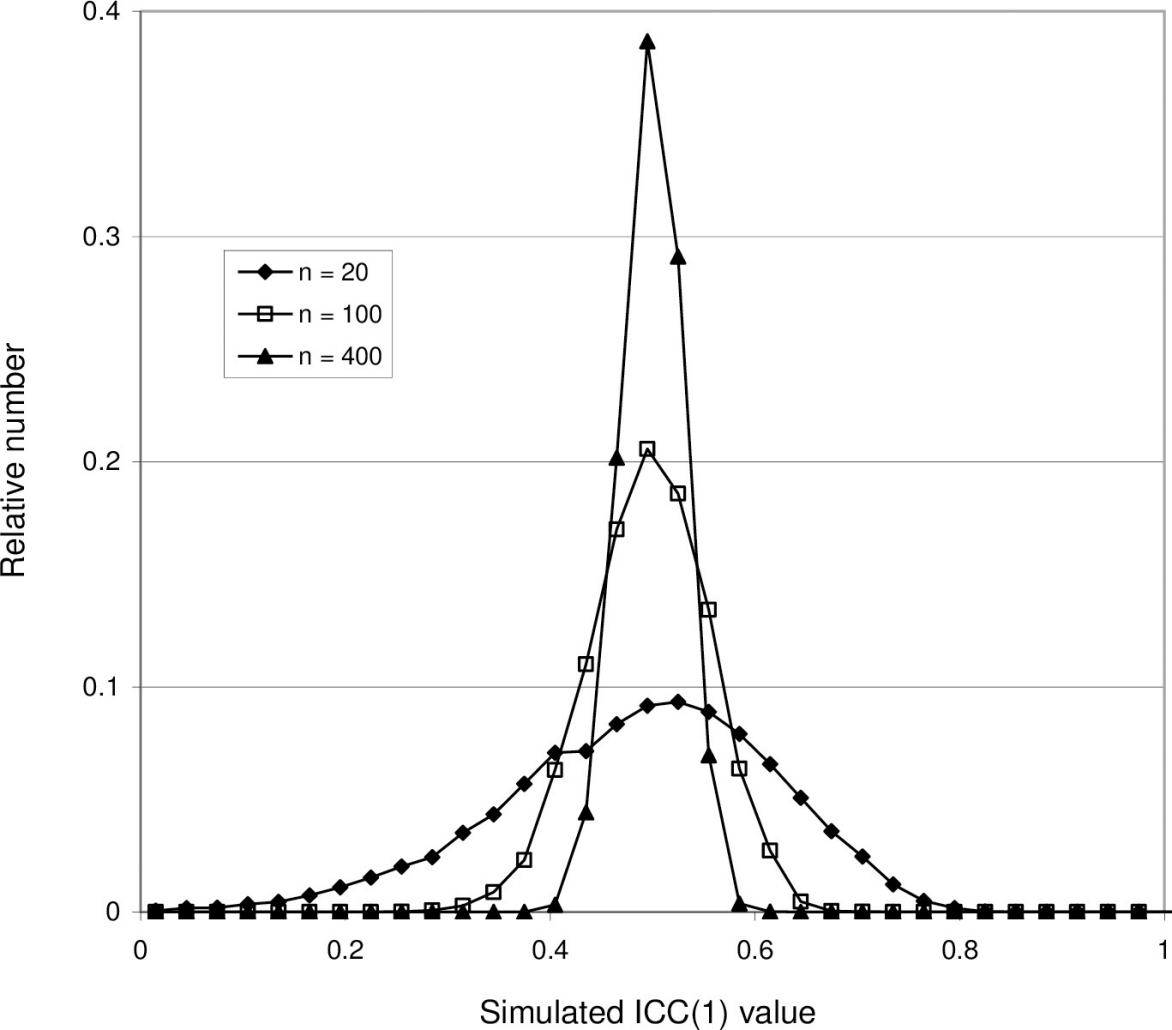
Probability distributions of ICC(1) obtained with Model 1, showing the effect of increasing the number of subjects. The number of subjects increases from *n* = 20 to *n* = 100 and *n* = 400. In all three cases *k* = 3 and the standard deviations of error and subject's score are 𝛔_*v*_ = *𝛔*_*r*_ = 10, giving the population ICC = 0.5. As can be seen, an increasing *n* leads to a decrease in the width of the probability distribution.

**Fig 7:**
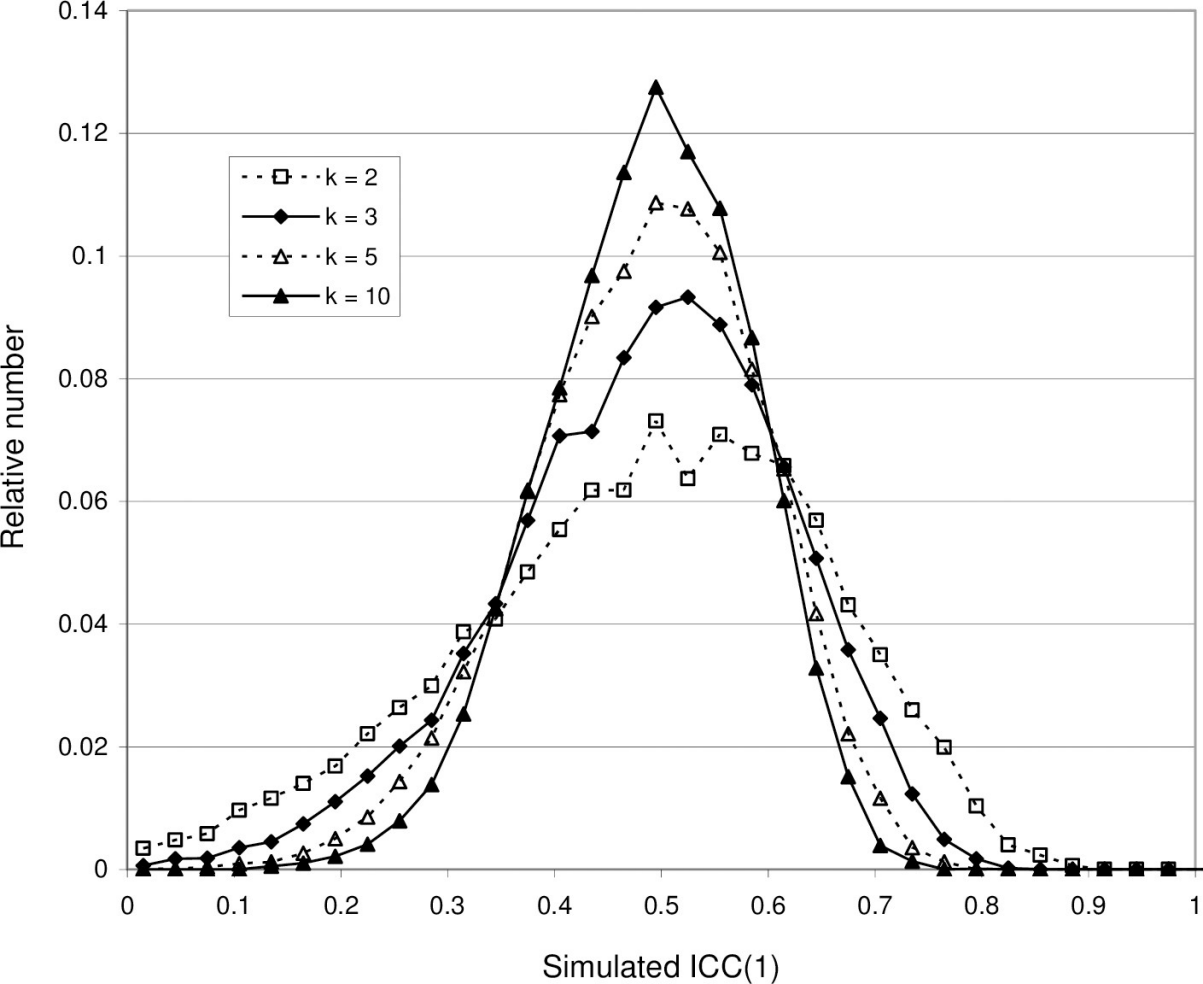
Probability distributions of ICC(1) obtained with Model 1, showing the effect of increasing the number of measurements *k*. In all four distributions, *n* = 20 and 𝛔_*v*_ *= 𝛔*_*r*_ = 10 (giving population ICC = 0.5). Increasing *k* leads to a decreasing width.

It is reasonable to expect that an increased number of subjects should lead to a better statistical accuracy. Fig 6 shows the effect of increasing *n* for the case ICC(1) = 0.5. The average value < ICC(1) > is not affected, but the ICC(1) distribution narrows, decreasing the 95% central range. Thus, the probability increases that the sample ICC will be close to the population ICC. A similar effect is obtained by increasing the number of measurements *k*, as shown by Fig 7. However, the number of measurements is for practical reasons usually limited, so the effect is less dramatic.

**Fig 8:**
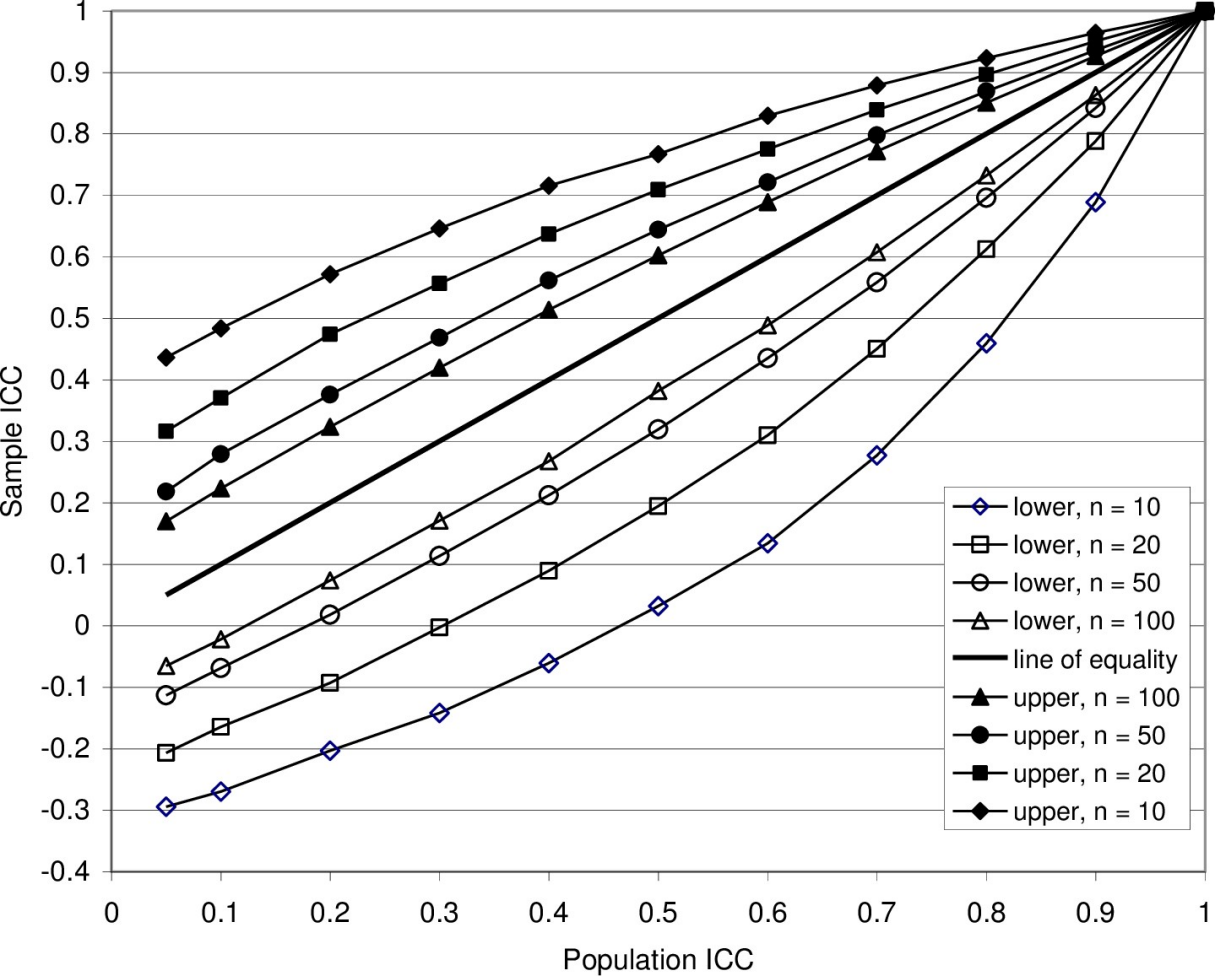
Graphic presentation of confidence limits. The curves show the upper and lower 95% central range limits of the ICC(1) probability distributions as functions of the population ICC, using Model 1. The number of measurements is everywhere *k* = 3 while the number of subjects *n* range from 10 to 100. Read horizontally, the diagram provides the 95% confidence limits of the population ICC for a given sample ICC(1) value. For example, if *n* = 10 and the sample ICC(1) is found to be 0.70, then the confidence limits of the population ICC are graphically read to be approximately 0.38 and 0.91. The diagram is also valid for ICC(C,1), i.e. the consistency ICC obtained with Model 2 and Model 3.

The 95% central range of ICC(1) is not the same thing as the confidence interval of *ρ*_1_, but the latter can be deduced from the former. In Fig 8 the curves show the upper and lower limits of the 95% central range of simulated ICC(1) distributions, for the cases *k* = 3 and *n* = 10, 20, 50 and 100, as functions of the population intraclass correlation coefficient *ρ*_1_ assumed in the simulation. To find the confidence intervals, the diagram may be used as follows.

Suppose, for example, that a single experimental matrix with *n* = 10, gives the sample value ICC(1) = 0.80. In Fig 8, the 95% lower and upper confidence limits of *ρ*_1_ are found where a horizontal line at ICC(1) = 0.80 crosses the *n* = 10 upper and lower 95% central range limits, i.e. (graphically) at *ρ*_1_ ≈ 0.55 and *ρ*_1_ ≈ 0.94. This is understood from the fact that, as can be seen from Fig 8, the probability of getting a sample ICC(1) = 0.8 if *ρ*_1_ < 0.55 is less than 2.5%; similarly, it is less than 2.5% if *ρ*_1_ > 0.94. With ICC(1) = 0.80 and *n* = 100, the confidence limits are in the same way graphically found to be *ρ*_1_ ≈ 0.74 and *ρ*_1_ ≈ 0.85.

This graphical method of estimating the ICC(1) confidence interval agrees well with the results obtained with the formulas given by McGraw and Wong [6] and the algorithms used in SPSS for Model 1. In fact, it should be pointed out that Monte Carlo simulation is in principle an exact method, apart from statistical "fuzziness". We will show that even in the presence of bias the graphical presentation in Fig 8 can be used for the consistency intraclass correlation coefficient ICC(C,1). However, it cannot be used for ICC(A,1). The graphical method (exemplified here only for *k* = 3) is not meant to replace confidence interval formulas [6] or, for example, SPSS software, but it demonstrates the connection with the ICC probability distributions as well as the general features of the confidence interval. Thus, Fig 8 shows that the confidence interval is centred approximately at the observed ICC value. Moreover, the confidence interval narrows with an increasing *n* and also with a higher sample ICC value. For low *n* and a low sample ICC value, it is however so wide that the population ICC may have almost any value below an upper limit.

An interesting point is worth mentioning. Suppose, for example, that the population ICC is *ρ*_1_ = 0.7 and that *n* = 10. Reading vertically, the 95% central range of the sample ICC probability distribution is found graphically to be approximately between 0.28 and 0.88 (Fig 8). For each sample ICC between these limits we obtain a certain confidence interval, as described above. Inspection of Fig 8 shows that one single population ICC value is common to all of these confidence intervals–namely *ρ*_1_ = 0.7. A moment of reflection shows that this result should be expected. The true (population) ICC is likely to be found in the overlap of any two 95% confidence intervals.

### 4.2. Simulation study of Model 2 (two-way random)

**Fig 9:**
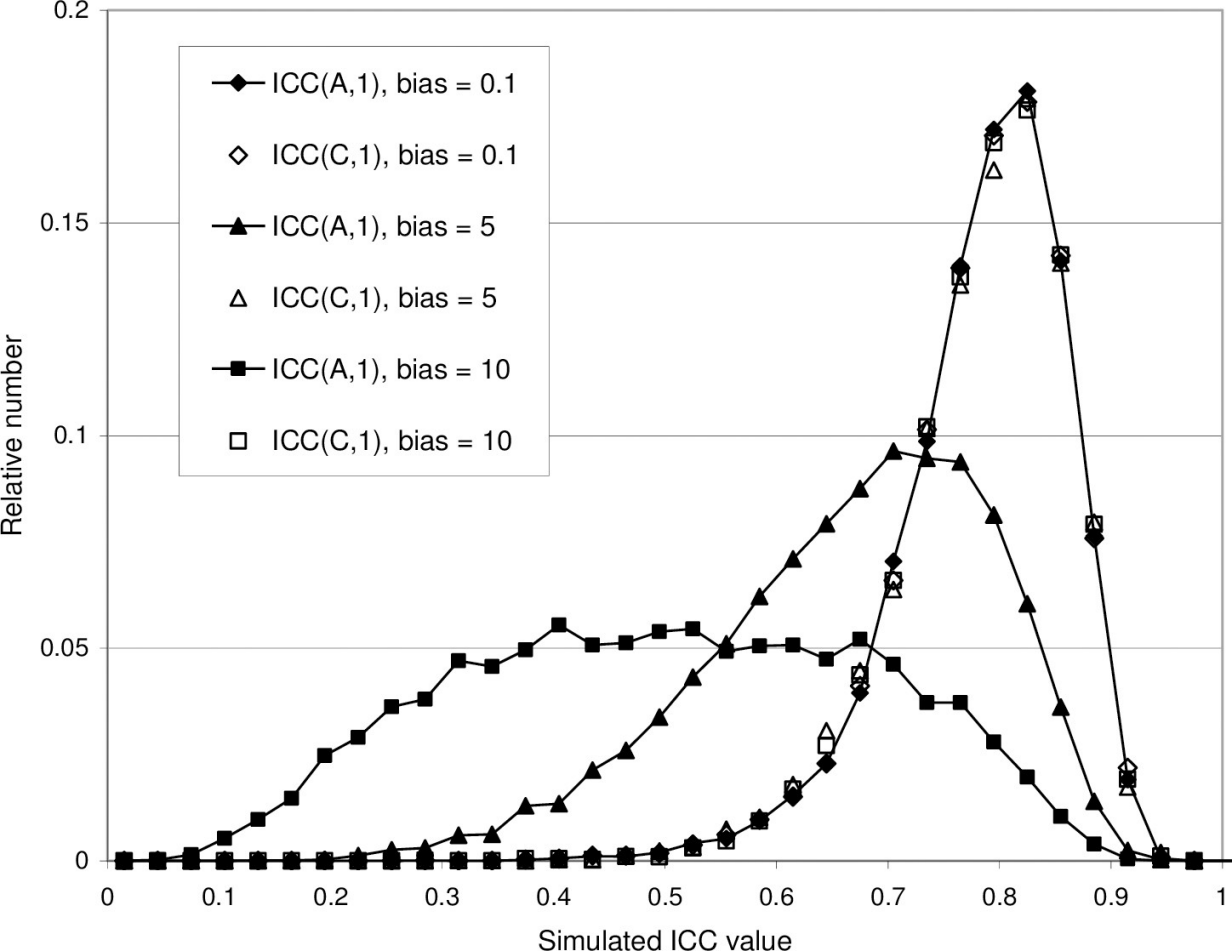
Probability distributions of ICC(A,1) and ICC(C,1) obtained with Model 2, showing the effect of increasing bias. In all cases *n* = 20, *k* = 3, *σ*_*r*_ = 10 and *σ*_*v*_ = 5. The bias standard deviation *σ*_*c*_ is increased from *σ*_*c*_ = 0.1 to *σ*_*c*_ = 5 and then to *σ*_*c*_ = 10. As may be seen, the ICC(C,1) distributions are insensitive to bias. The ICC(A,1) distributions are, with increasing bias, shifted towards lower values and broadened.

As discussed in Section 2.4, the only difference between Model 1 and Model 2 is the presence, in Model 2, of bias (systematic error) terms *c*_*j*_ sampled from a normal distribution with standard deviation *σ*_*c*_ and mean value = zero. To see the effect of such biases, we may therefore start with Model 1, i.e. *σ*_*c*_ = 0, and then turn on a non-zero bias, sampling the *c*_*j*_ from normal distributions with successively larger bias variance *σ*_*c*_^2^ (keeping everything else fixed). This is demonstrated in Fig 9 and Table 3.

**Table 3:** A survey of features of Model 2.

Model 2	<ICC(A,1)> (95% central range ICC(A,1) distribution) <ICC(1)>					<ICC(C,1)> (95% central range)
	*σ*_*c*_ /*σ*_*r*_ (relative bias)					
*σ*_*v*_ */ σ*_*r*_ (relative noise)	0	0.1	0.5	1.0	1.5	(independent of bias)
0.1	0.99 (0.98–1.00) 0.99	0.98 (0.94–0.99) [0.94]	0.81 (0.46–0.99) [0.78]	0.58 (0.19–0.97) [0.49]	0.43 (0.09–0.94) [0.26]	0.99 (0.98–1.00)
0.5	0.78 (0.61–0.90) 0.78	0.78 (0.60–0.89) [0.78]	0.67 (0.37–0.86) [0.64]	0.50 (0.16–0.83) [0.41]	0.38 (0.08–0.78) [0.22]	0.79 (0.60–0.90)
1.0	0.48 (0.20–0.71) 0.48	0.48 (0.19–0.71) [0.48]	0.44 (0.16–0.68) [0.41]	0.35 (0.10–0.63) [0.28]	0.28 (0.06–0.59) [0.14]	0.48 (0.19–0.71)
1.5	0.30 (0.00–0.57) 0.30	0.30 (0.00–0.56) [0.30]	0.28 (0.00–0.54) [0.26]	0.24 (0.00–0.50) [0.18]	0.20 (0.00–0.47) [0.09]	0.30 (0.00–0.57

Simulated results are shown for *n =* 20 and *k =* 3. In columns 2–6 is shown the average ICC(A,1) value, the 95% central range (within parenthesis) of the simulated ICC(A,1) distribution, and the average ICC(1) value. The latter is shown within brackets where the ICC(1) formula is invalid. Column 7 shows the average value and 95% data range of the ICC(C,1) distribution. The data may be compared with probability distributions shown in Fig 5 and Fig 9.

Fig 9 shows the distributions of ICC(A,1) and ICC(C,1) obtained with three different versions of Model 2, namely with a "weak" bias (*σ*_*c*_*/σ*_*r*_ = 0.01), a "moderate" bias (*σ*_*c*_*/σ*_*r*_ = 0.5), and a "strong" bias (*σ*_*c*_*/σ*_*r*_ = 1.0). In the "weak" bias case, the results of Model 2 are practically identical to the results of Model 1 (*σ*_*c*_*/σ*_*r*_ = 0)–at least with the modest number of subjects *n* = 20. As can be seen from Fig 9, the effect of an increasing bias *σ*_*c*_ is a lower average value <ICC(A,1)> and a wider ICC(A,1) probability distribution. The average ICC value in each distribution agrees approximately with the population ICC. Using Eq (9) we find *ρ*_2A_ = 10^2^/(10^2^ + 0.1^2^ + 5^2^) ≈ 0.80, *ρ*_2A_ = 10^2^/(10^2^ + 5^2^ + 5^2^) ≈ 0.67 and *ρ*_2A_ = 10^2^/(10^2^ + 10^2^ + 5^2^) ≈ 0.44 for the three ICC(A,1) distributions, respectively. The increased width of the ICC(A,1) distributions with increasing *σ*_*c*_ may be attributed to the increasing contribution from the random variation of the *c*_*j*_ terms.

The ICC(C,1) distributions are insensitive to the presence of bias; they remain the same, regardless of the strength of the bias. Using Eq (13) we find *ρ*_2C_ = 10^2^/(10^2^ + 5^2^) = 0.8 for all three ICC(C,1) distributions. They coincide with the ICC(1) (zero bias) distribution as well as with the ICC(A,1) distribution when biases are very small. This indicates that the confidence limits of *ρ*_*2C*_ (the consistency population ICC of Model 2) will be the same as the confidence limits of *ρ*_1_ (population ICC of Model 1, i.e. in the absence of bias). Thus, Fig 8 may be used to graphically derive not only the confidence limits corresponding to an ICC(1) value calculated from an experimental matrix compatible with Model 1, but also the confidence limits corresponding to an ICC(C,1) value calculated from a matrix compatible with Model 2. This observation is in agreement with the confidence limit formulas given by McGraw and Wong [6] provided that the number of subjects *n* is not too small.

Since we may turn the bias down all the way to zero without changing the ICC(C,1) distribution, ICC(C,1) may, as already discussed in Section 2.5.3, be regarded as an estimate of the intraclass correlation that would be obtained if the effects of bias could be removed or corrected for [17]. However, it should also be observed that the ICC(C,1) value might give an exaggerated and misleading impression of the reliability of a method unless it is clearly stated that the value given is the consistency ICC value.

Table 3 gives an survey of the main features of Model 2 for the case *n* = 20, *k* = 3, showing the average values < ICC(A,1) > and < ICC(C,1) > as well as the 95% central range limits obtained from the simulated distributions. The average values and central range limits are shown as functions of the two parameters (*σ*_*v*_/*σ*_*r*_) and (*σ*_*c*_/*σ*_*r*_) (see Eq (36)). The average values of ICC(1) are for comparison shown also for *σ*_*c*_ > 0, even though the formula ICC(1) is then invalid. It may be noted that excellent ICC(A,1) values are with any certainty only found in the upper left corner; relative noise as well as relative bias have to be at most about 0.1. Occasionally, a high ICC(A,1) value could be obtained even with a strong relative bias; the 95% central range is very wide. The results for ICC(C,1) are, as already discussed, independent of *σ*_*c*_ and equal to the results obtained for ICC(1) with Model 1. Average invalid ICC(1) values (in brackets) are everywhere lower than the ICC(A,1) and ICC(C,1) values. For *σ*_*c*_ = 0 (Model 1) we find < ICC(1) > = < ICC(A,1) > = < ICC(C,1 >. We also find equal 95% central range limits, i.e. equal distributions as also exemplified in Fig 4.

Table 4 gives an example (for *n* = 20 and *k* = 3) of what to expect when the ratio ICC(C,1)/ICC(A,1), where ICC(C,1) and ICC(A,1) have been calculated from the same matrix, is used as a qualitative indication of the presence of bias. The simulated average ratio <ICC(C,1)/ICC(A,1)> obtained with Model 2 is shown, as well as the probability of finding an ICC(C,1) value larger than ICC(A,1). The average ratio increases with increasing bias and decreases with increasing noise. In the absence of bias the average ratio is very close to unity; the (very narrow) distribution is such that it is actually more probable to find an ICC(C,1) value that is lower than ICC(A,1). With a low relative bias (≈ 0.1) the difference between ICC(C,1) and ICC(A,1) is seen to be of order 1%. With a high relative bias (≈ 1) it might be as much as a factor ≈ 2. As mentioned, it is strongly recommended to use an F-test as well (see Section 2.4.5).

**Table 4:** Survey of model 2 for n = 20 and k = 3: the average ratio <ICC(C,1)/ICC(A,1)> and the probability for ICC(C,1) to be larger than ICC(A,1) in a single data matrix.

Model 2 *n* = 20, *k* = 3	< ICC(C,1) / ICC(A,1) > (Probability of ICC(C,1) > ICC(A,1))				
	*σ*_*c*_ /*σ*_*r*_ (relative bias)				
*σ*_*v*_ */ σ*_*r*_ (relative noise)	0	0.1	0.5	1.0	1.5
0.1	1.000 (37%)	1.011 (95%)	1.278 (99.8%)	2.114 (100%)	3.506 (100%)
0.5	1.000 (37%)	1.009 (58%)	1.215 (96%)	1.857 (99%)	2.947 (99%)
1.0	1.001 (38%)	1.006 (44%)	1.134 (85%)	1.526 (95%)	2.165 (98%)
1.5	1.001 (38%)	1.005 (42%)	1.080 (73%)	1.318 (91%)	1.727 (95%)

Shown in each cell in columns 2–6 is the simulated average ratio < ICC(C,1) / ICC(A,1) > where ICC(C,1) and ICC(A,1) have been calculated from the same matrix. Within parenthesis is shown the probability that the ICC(C,1) value will be larger than the ICC(A,1) value.

### 4.3. Simulation study of Model 3 (two-way mixed model)

In order to demonstrate features of Model 3, we will discuss the following example. Let us assume that there are two research groups, A and B, each consisting of *k* = 3 raters. These two groups intend to carry out the same kind of measurements on the same population (with *σ*_*r*_ = 10), and for this purpose each of them makes a reliability study on *n* = 20 subjects (not the same subjects).

For the present argument we will make a comparison with Model 2. We assume that the raters in each group have been randomly chosen from a population of raters normally distributed with a bias standard deviation *σ*_*c*_ = 5. The probability distributions of all possible ICC(A,1) and ICC(C,1) values in a reliability study with such raters are given by Model 2 and shown in Fig 9, where they are marked "bias = 5".

Assume that each group has made a reliability study. We now consider the fact that each group, A and B, has a certain set of biases *c*_1_, *c*_2_ and *c*_3_. We assume that group A has a set (a) of biases, namely *c*_1_ = 1, *c*_2_ = 6 and *c*_3_ = -1, while group B has a set (b) of biases *c*_1_ = 10, *c*_2_ = 6 and *c*_3_ = - 10. The set (a) is not unlikely, while the set (b) is very improbable but still possible.

**Fig 10:**
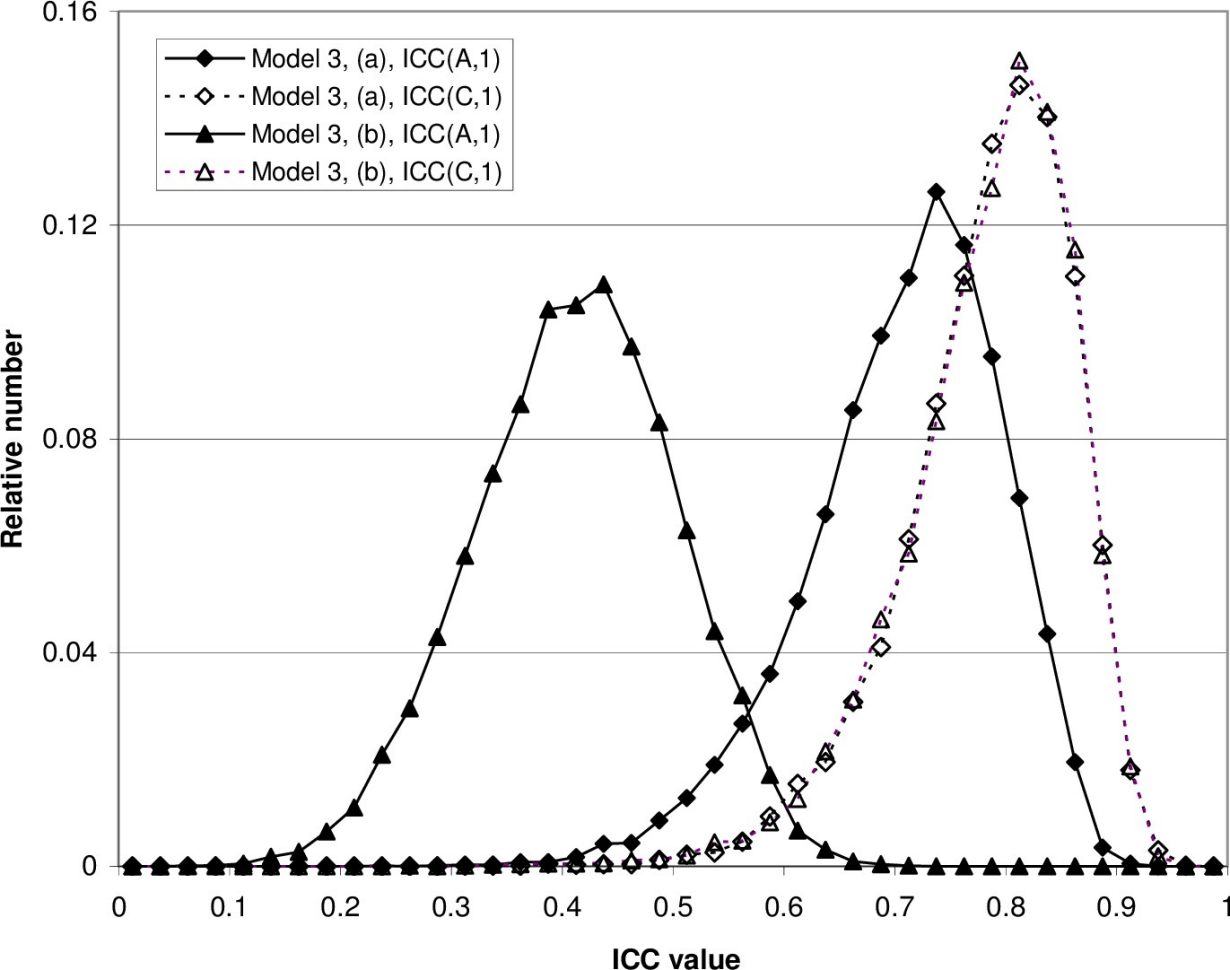
Effect of fixed bias: ICC(A,1) and ICC(C,1) distributions simulated using Model 3. Two simulated Model 3 cases are shown, (a) and (b). In both cases, *n* = 20, *k* = 3, *σ*_*r*_ = 10 and *σ*_*v*_ = 5. In case (a), the fixed bias values are *c*_1_ = 1, *c*_2_ = 6 and *c*_3_ = -1. In case (b), they are *c*_1_ = 10, *c*_2_ = 6 and *c*_3_ = -10. A larger spread among the fixed bias values gives a larger shift of the ICC(A,1) distribution towards lower values. The ICC(C,1) distributions are however insensitive to bias.

Using Model 3 (simulation with fixed biases), these two sets of biases have been used to simulate the two ICC(A,1) and two ICC(C,1) probability distributions shown in Fig 10. We may note that the ICC(C,1) distributions again coincide with each other and also with the ICC(C,1) distributions in Fig 9.

The ICC(A,1) probability distributions in Fig 10 are however distinctly different from each other. They are the probability distributions from which group A and group B will actually obtain their ICC(A,1) values in a reliability study, if their biases are regarded as given. Thus, they are also the probability distributions for the ICC(A,1) values that would be obtained in a second reliability study. What Fig 10 shows is simply that if group A and group B would repeat their respective reliability studies, they would probably obtain an ICC(A,1) value not far from the one found in their first study. With a third set C of three randomly selected raters we may of course expect another result, as indicated by the Model 2 distribution in Fig 7.

We may understand the details of the Model 3 distributions in Fig 10 as follows. In case (a), the bias values *c*_1_ = 1, *c*_2_ = 6 and *c*_3_ = -1 give, using Eq (23), *θ* ^2^ = 13. Using Eq (24), the absolute agreement population ICC is then *ρ*_3A_ = 10^2^/(10^2^ + 13 + 5^2^) ≈ 0.725. Using Eq (27), the consistency population ICC is *ρ*_3C_ = 10^2^/(10^2^ + 5^2^) = 0.8. We can see from Fig 10 that the maxima of the ICC(A,1) and ICC(C,1) distributions in case (a) are positioned near these *ρ*_3A_ and *ρ*_3C_ values. The width of the ICC(A,1) distribution obtained with Model 3 is somewhat smaller than the width of the ICC(A,1) ("bias = 5") distribution obtained with Model 2 in Fig 9, reflecting the fact that since the *c*_*j*_ are fixed, the width is due only to the variation in the subject scores *r*_*j*_ and the noise *v*_*ij*_.

In case (b), the fixed bias values *c*_1_ = 10, *c*_2_ = 6 and *c*_3_ = -10 give in the same way *θ* ^2^ = 112, from which the absolute agreement population ICC is found to be *ρ*_3A_ ≈ 0.422. The consistency population ICC is again found to be *ρ*_3C_ = 0.8 and again the ICC(A,1) and ICC(C,1) distributions are approximately centred on these values.

Thus, when the choice of raters has been made, it may turn out that the ICC(A,1) values obtained by the two research groups A and B are very different from each other, due to their different biases. This is more likely to happen if the number of raters in each group is small and the raters have been sampled from a population with a large standard deviation *σ*_*c*_.

In general we should expect that the ICC(A,1) 95% confidence intervals obtained by group A and group B will overlap. Within this overlap we may, as indicated in Section 4.1, expect to find the Model 2 population intraclass coefficient *ρ*_*2A*_.

## 5. Analysis procedure

### 5.1. Conventional approach

In the conventional approach to the analysis of a reliability study it is common to specify one statistical model (the one-way random, the two way random or the two-way mixed model) in accordance with the design of the study [2,6]. For example, in the SPSS software it is required that the one-way random model is chosen if the ICC(1) value is to be calculated. Likewise, if the ICC(A,1) value is desired, one has to choose the absolute agreement two-way random or two-way mixed model. To obtain the ICC(C,1) value, one has to choose the consistency two-way random or two-way mixed model. Similar recommendations are given in the flow charts presented by McGraw and Wong [6] and Koo et al [2].

### 5.2. Present approach

We find that the initial specification of a statistical model is inconvenient and not necessary. Of course, the researcher may, from the design of a reliability study, expect that a certain model should be appropriate (see Section 2.6). Reasonably, the design has been such that it might be hoped that not only error (noise) but also systematic differences between measurements (bias) are negligible, in which case Model 1 (one-way random) can be used. However, it may turn out that the model has to be reconsidered. Unexpected biases may turn up; also, the effect of biases may be larger than foreseen.

We therefore propose that while the design of the method should, of course, be planned with utmost care, the analysis of the result of the reliability study should be impartially open to all different possibilities. Our recommendation is similar to that previously given by Weir [19].

The analysis procedure may for example be as follows. We start by calculating the mean squares *MS* from the matrix of experimental data and, from them, the sample ICC values (ICC(1), ICC(A,1) and ICC(C,1), using the standard formulas shown e.g. in Fig 1. If we find that ICC(1) ≈ ICC(A,1) ≈ ICC(C,1), i.e. approximately equal (within a few percent; see Table 4), then biases are likely to be small or even negligible. If an F-test with *F = MSBM/MSE* shows that Model 1 cannot be rejected, then ICC(1) may be reported as a result, with the comment that biases are negligible. We may note, though, that all three ICC values may then be regarded equally valid estimates of the population intraclass coefficient. The estimated magnitude of the spread in true scores and the noise are obtained by calculating the standard deviations *σ*_*r*_ and *σ*_*v*_, using Eq (5).

Distinctly different ICC(1), ICC(A,1) and ICC(C,1) values from the same matrix indicate the presence of non-negligible biases (see Table 4), i.e. systematic differences between different measurements *j* = 1,2, …,*k*. Usually, one then finds ICC(1) < ICC(A,1) < ICC(C,1); however, ICC(1) is no longer a valid formula. The *F* test may be used to definitely reject Model 1, confirming the probable presence of bias. We need not specify whether Model 2 (two-way random) or Model 3 (two-way mixed) is used instead of Model 1; it makes no difference.

Thus, we avoid the error of reporting the ICC(1) value in the presence of bias. We report instead two ICC values, ICC(A,1) and ICC(C,1), together with their confidence intervals and a comment on the presence of bias. The estimated magnitudes of spread in true scores and of bias and noise are obtained by calculating the standard deviations *σ*_*r*_, *σ*_*v*_ and *σ*_*c*_, using Eq (11).

### 5.3. A clinical example

To exemplify the analysis procedure we use clinical data from Elfving et al [20]. The measured data, shown in Table 5, have been extracted from a reliability study of a method using electromyography. The data are a subset of electromyographic recordings made on back muscles on three separate days, namely measurements made in the morning on 10 subjects.

**Table 5:** A clinical example from physiotherapy.

Subject	Day 1	Day 2	Day 3	Mean
1	59.9	67.7	72.2	66.6
2	62.9	66.5	67.9	65.8
3	58.9	50.1	47.9	52.3
4	46.8	50.0	53.9	50.3
5	62.5	67.8	62.6	64.3
6	44.8	42.7	48.4	45.3
7	57.3	49.6	48.0	51.7
8	49.0	45.2	57.5	50.6
9	43.5	41.5	47.4	44.2
10	39.2	50.9	56.3	48.8
**Mean**	52.5	53.2	56.2	54.0

Electromyographic median frequency data (Hz) obtained from the lumbar left side of the back muscles, recorded on three separate days (from Elfving et al 1999).

For the present analysis we have used SPSS version 24 as a calculation tool. Application of ANOVA (as part the ICC calculation in SPSS) gave the mean squares *MSBS* = 212.61, *MSBM* = 39.15, *MSE* = 24.45 and *MSWS* = 25.92. Results for single-score ICC with 95% confidence intervals were, choosing the one-way random model, ICC(1) = 0.706 (0.387–0.906) and, choosing the two-way random model, ICC(A,1) = 0.708 (0.392–0.907) and ICC(C,1) = 0.720 (0.396–0.912). We may note that the confidence interval obtained for ICC(1) and ICC(C,1) agrees well with a graphical reading from Fig 8. It is clear from Fig 8 that somewhat less wide confidence intervals could have been obtained with a larger number of subjects. We need not use the two-way mixed model; it gives exactly the same results as the two-way random model.

We observe that ICC(1), ICC(A,1) and ICC(C,1) are closely equal (as are also their confidence intervals), which suggests that there are no systematic differences between the recordings from day to day, i.e. no bias; thus ICC(1) might be correctly reported. ICC(C,1) is slightly higher which indicates that there might possibly be a weak bias; the ratio ICC(C,1)/ICC(A,1) is 1.017 (compare Table 4). To make more certain, we look at the between days *F*-value which is *F* = *MSBM/MSE* = 1.601 with *p* = 0.229. This low F-value and relatively high *p* value shows that the systematic differences between days are not significant; i.e. the one-way random model (no bias) cannot be rejected.

Additional insight is provided by calculating the estimated standard deviations. Assuming first that the one-way model is applicable we find manually, using the mean squares quoted above and Eq (5), the standard deviations *σ*_*r*_ = 7.89 Hz and *σ*_*v*_ = 5.09 Hz. Thus, even if biases are assumed to be absent, the error *σ*_*v*_ is substantial, i.e. 5.09/7.89 = 65% of the spread *σ*_*r*_ among the true scores of the subjects. To discern the magnitude of a possible bias, we use instead the two-way random model and apply Eq (11), which, with the mean squares quoted above, gives *σ*_*r*_ = 7.92 Hz, *σ*_*v*_ = 4.94 Hz and *σ*_*c*_ = 1.21 Hz. Thus, if biases exist, they are small, and the F-test has already told us that the one-way model cannot be rejected. It may be noted that both *σ*_*v*_ values are estimates of the standard error of measurement, one (5.09 Hz) obtained from *MSWS* and the other (4.94 Hz) from *MSE*. The standard error of measurement in connection with ICC is discussed by for example [19] and [32].

However, even if there were no significant biases, the result was not encouraging. The ICC value obtained, ICC ≈ 0.71, was not considered to be good enough for the intended use of the method as a diagnostic tool [20].

## 6. Discussion

Of course, a basic aim when designing a method of measurement should be to have small random errors and negligible systematic errors. Indeed, if the precise value of a measurement is important we cannot afford to have anything else. A good outcome of a reilability study would therefore be to find that Model 1 (the one-way random model) is valid and that the ICC(1) value is good or excellent; or, more precisely, that the lower limit of the confidence interval of ICC(1) is found to be in the range from good to excellent.

Unfortunately, this is not always the case. For example, while systematic errors might be negligible, random errors may be too large, as in the clinical exemple just described.

If, on the other hand, biases are non-negligible but expected to be randomly varying (Model 2), is the method then reliable? That should depend on its intended use. Model 2 means that in another measurement biases will usually be different, and therefore it will in general not be possible to correct for them. The method might still be reliable to a limited extent as regards absolute agreement, provided that the randomly varying biases are not too large. However, from Table 3 it can be seen that for relative biases ≥ 0.5, one should hardly expect an ICC(A,1) value better than moderate or good.

If ICC(C,1) is found to be good or excellent, but ICC(A,1) is poor or moderate, then the method might still be used to obtain information on consistency (ranking order and differences between subjects). The presence of noise (random error) will limit this restricted reliability of the method. Since ICC(C,1) is insensitive to the presence of bias, results as regards consistency made by different raters or research groups should be possible to compare.

If ICC(C,1) as well as ICC(A,1) are poor, then the method of measurement is of course not reliable at all and should be re-designed or discarded.

Finally we should discuss Model 3 (two-way mixed model). We have already seen that in the analysis of a reliability study it may seem meaningless to differ between Model 2 and Model 3 since they give equal results.

Still, we wish to examine what difference there is. We therefore consider the Model 2 and Model 3 definitions of the ICC. Formally, they differ only in that the term *σ*_*c*_ in the Model 2 definition, Eq (9), has been replaced by the term *θ*_*c*_ in the Model 3 definition, Eq (24). From the derivation of the EMS relations (S2 Appendix using Eq (A2-6) and Eq (A2-8)) we find that in Model 2

$$\sigma_{c}{}^{2}\approx \frac{\sum_{j}{\left(c_{j}-\bar{c}\right)}^{2}}{\left(k-1\right)}$$ (39)

while in Model 3

$$\theta_{c}{}^{2}=\frac{\sum_{j}{\left(c_{j}-\bar{c}\right)}^{2}}{\left(k-1\right)}$$ (40)

Comparing Eq (39) and Eq (40), we can see that the meaning of Eq (39) is that the quantity *θ*_*c*_^2^ calculated by Eq (40) is assumed to be an estimate (≈) of the variance *σ*_*c*_^2^ of a normal distribution of biases (e.g., raters) from which these biases *c*_*j*_ have been sampled. In Model 3 one has evidently refrained from making this assumption.

Now, the ICC is basically defined as the variance (regarded as a measure of variation) of interest, divided by the total variance. However, fixed *c*_*j*_ values do not represent a variation; for example, simulations using Model 3 give ICC distributions that are narrower than those obtained with Model 2, as seen by comparing Fig 9 and Fig 10. Therefore, one might hesitate about the meaning and justification of the term *θ*_*c*_^2^ in Eq (24). Removing it, one obtains the consistency intraclass correlation Eq (27). Indeed, when biases are fixed the use of the consistency intraclass correlation is the only option considered by Shrout and Fleiss [5].

Nevertheless, *θ*_*c*_^2^ is present in Eq (24). We conclude that *θ*_*c*_^2^ in Eq (24) is in fact equivalent to an estimate of the variance of a population of biases. In other words, as regards evaluating ICC(A,1) from a study of reliability, Model 3 is not only in practice but also conceptually equivalent to Model 2. In the practical analysis of a reliability study it will therefore be sufficient to consider Model 1 and Model 2 (see the clinical example in Section 5.3).

A statement of fixed biases is thus not relevant for the calculation of ICC, but may be regarded as information about how the researcher intends to proceed [6]. Fixed biases should mean that the researcher intends to continue with the same method, staff and experimental set-up, so that the biases (if any) may be expected to remain the same. One should of course not expect an absolute agreement between experimental set-ups with different fixed biases.

If the fixed biases are substantial (i.e. if ICC(A,1) is poor or moderate), it seems that a reasonable approach should be to try to correct for or remove them. If that is not possible, ICC(C,1) still gives an estimate of the reliability of results as regards consistency, i.e. ranking order and differences between subjects. Results as regards consistency obtained by different research groups may be possible to compare.

## 7. Conclusion

Our main, practical conclusion is as follows. It is not necessary and indeed inconvenient to be locked to a definite statistical model (one-way random, two-way random or two-way mixed) when beginning the analysis of a matrix of single-score experimental data obtained in for example a reliability study. All three single-score intraclass correlation coefficient versions, i.e. ICC(1) (classical), ICC(A,1) (absolute agreement) and ICC(C,1) (consistency) may be calculated and compared. A close agreement between them (within a few percent) indicates qualitatively the absence of systematic error (biases), while disagreement, with an ICC(C,1) value larger than ICC(A,1), indicates the presence of bias. An F-test will show if the null hypothesis (absence of bias) should be rejected. If not, the ICC(1) value may be reported together with its confidence interval. However, in the presence of bias, ICC(1) is no longer a valid formula and should not be used. ICC(A,1) as well as ICC(C,1) are valid in the presence as well as in the absence of measurement bias. The two coefficients provide different and complementary information about the reliability of the method and both should be reported together with their confidence intervals. Thus, ICC(A,1) provides an estimate of the reliability where the effect of bias is taken into account, while ICC(C,1) neglects this effect. As previously suggested, ICC(C,1) may be said to provide an estimate of the ICC that would be obtained if biases were absent.

## Supporting information

S1 Appendix ANOVA. Sum of squares and mean squares–definitions and relations.
(PDF)

S2 Appendix Derivation of expected mean square relations. (PDF)

S3 Appendix Monte Carlo sampling. Sampling from a normal distribution by means of the Monte Carlo method.
(PDF)

S4 Appendix Simulation program. Fortran 77 code SIMANOVA.
(FOR)
