# Supplementary material for: Intraclass correlation – A discussion and demonstration of basic features
Source: PLoS One. 2019 Jul 22;14(7):e0219854. doi: 10.1371/journal.pone.0219854 (PMC6645485; doi:10.1371/journal.pone.0219854)
Supplement: S1 Appendix — Sum of squares and mean squares–definitions and relations. (PDF) [file pone.0219854.s001.pdf]

Appendix 1
ANOVA sum of squares and mean squares – definitions and relations

We refer to Table 2, main text. Each row (i = 1, ... ,n) of the matrix normally corresponds to a "subject", while each column (j = 1, ... ,k) corresponds to a "measurement", "rater" or "condition", depending on the context. We have chosen the word "subject" for rows, and "measurement" for columns.

First, we define the mean value S_i for each subject (row) i, the mean value M_j for each measurement (column) j, and the total mean value $\bar{x}$ of all the measured values x_{ij}.

$$S_i = \frac{1}{k}\sum_{j=1}^{k} x_{ij} \qquad \text{(A1-1)}$$

$$M_j = \frac{1}{n}\sum_{i=1}^{n} x_{ij} \qquad \text{(A1-2)}$$

$$\bar{x} = \frac{1}{n \cdot k}\sum_{i=1}^{n}\sum_{j=1}^{k} x_{ij} \qquad \text{(A1-3)}$$

The various sums of squares may now be defined in a symmetrical way by double sums:

$$SST = \sum_{i=1}^{n}\sum_{j=1}^{k}(x_{ij} - \bar{x})^2 \qquad \text{(A1-4)}$$

(Sum of Squares, Total)

$$SSBS = \sum_{i=1}^{n}\sum_{j=1}^{k}(S_i - \bar{x})^2 \qquad \text{(A1-5)}$$

(Sum of Squares Between Subjects)

$$SSBM = \sum_{i=1}^{n}\sum_{j=1}^{k}(M_j - \bar{x})^2 \qquad \text{(A1-6)}$$

(Sum of Squares Between Measurements)

$$SSWS = \sum_{i=1}^{n}\sum_{j=1}^{k}(x_{ij} - S_i)^2 \qquad \text{(A1-7)}$$

(Sum of Squares Within Subjects)

$$SSWM = \sum_{i=1}^{n}\sum_{j=1}^{k}(x_{ij} - M_j)^2 \qquad \text{(A1-8)}$$

(Sum of Squares Within Measurements)

$$SSE = SST - SSBS - SSBM \qquad \text{(A1-9)}$$
(Sum of Squares, Error)

We have here for convenience expressed *SSE* (often called "residual" instead of "error") as the difference between *SST* and (*SSBS+SSBM*), although it may, like the others, be defined by means of a double summation. From the definitions (A1-4) – (A1-9) the following exact relation may be derived:

$$SST = SSBS + SSWS = SSBM + SSWM \qquad \text{(A1-10)}$$

This may be used together with (A1-9) to derive other useful relations, for example

$$SSWM = SSBS + SSE \qquad \text{(A1-11)}$$

$$SSWS = SSBM + SSE \qquad \text{(A1-12)}$$

From the sums of squares the mean squares (MS) are calculated as follows:

$$MST = \frac{SST}{n \cdot k - 1} \qquad \text{(A1-13)}$$
(Mean Square, Total)

$$MSBS = \frac{SSBS}{n - 1} \qquad \text{(A1-14)}$$
(Mean Square Between Subjects)

$$MSBM = \frac{SSBM}{k - 1} \qquad \text{(A1-15)}$$
(Mean Square Between Measurements)

$$MSWS = \frac{SSWS}{n \cdot (k - 1)} \qquad \text{(A1-16)}$$
(Mean Square Within Subjects)

$$MSWM = \frac{SSWM}{k \cdot (n - 1)} \qquad \text{(A1-17)}$$
(Mean Square Within Measurements)

$$MSE = \frac{SSE}{(n-1) \cdot (k-1)} \qquad \text{(A1-18)}$$
(Mean Square, Error)

The denominators in (A1-13) – (A1-18) are the respective degrees of freedom (df).
