# Supplementary material for: Intraclass correlation – A discussion and demonstration of basic features
Source: PLoS One. 2019 Jul 22;14(7):e0219854. doi: 10.1371/journal.pone.0219854 (PMC6645485; doi:10.1371/journal.pone.0219854)
Supplement: S2 Appendix — (PDF) [file pone.0219854.s002.pdf]

Appendix 2
Derivation of the EMS (Expected Mean Square) relations

For this derivation we use a matrix with n = 3 rows (subjects) and k = 2 columns (measurements) as an illustration:

x_{11}	x_{12}	S_1
x_{21}	x_{22}	S_2
x_{31}	x_{32}	S_3
M_1	M_2	$\bar{x}$

As in Table 2 (main text), S_1, S_2, and S_3 are the mean values of the rows, M_1 and M_2 are the mean values of the columns and $\bar{x}$ is the total mean value.

We will first assume Model 2, i.e. that each matrix element x_{ij} may be regarded as the sum of four terms,

$$x_{ij} = \mu + r_i + c_j + v_{ij} \qquad \text{(A2-1)}$$

where μ is a constant, r_i is sampled from a normal distribution with standard deviation σ_r, c_j is sampled from a normal distribution with standard deviation σ_c and v_{ij} is sampled from a normal distribution with standard deviation σ_v. Note that Model 1 is obtained simply by putting all $c_j = 0$.

Assuming that the model (A2-1) is used to generate the matrix x_{ij} shown above, we will now estimate the resulting mean squares, i.e. *MSBS*, *MSBM*, *MSWS*, *MSWM* and *MSE*.

We observe that each matrix element x_{ij} is, apart from the constant μ, the sum of three terms, each of which is sampled from a normal distribution. The total variance of x_{ij} is therefore the sum of the three independent variances $\sigma_r^{\ 2}$, $\sigma_c^{\ 2}$ and $\sigma_v^{\ 2}$. It follows that each term in (A2-1) will give its own, independent contribution to each of the above five mean squares, for example to *MSBS*. In order to estimate these contributions, the simplest procedure is to study one term in (A2-1) at a time, assuming the others to be zero.

We may expect the constant μ to give zero contribution to variance and thus zero contribution to each mean square quantity (*MS*). This is easily verified. Putting each term in eq.(A2-1) except μ equal to zero, the matrix and its averages in will be reduced to the following:

μ	μ	μ
μ	μ	μ
μ	μ	μ
μ	μ	μ

We use the formulas in Appendix 1, and get, for example,

$$MSBS = \frac{SSBS}{n-1} = \frac{\sum_{i,j}(S_i - \bar{x})^2}{n-1} = \frac{\sum_{i,j}(\mu - \mu)^2}{n-1} = 0 \qquad \text{(A2-2)}$$

In a similar way we may easily confirm that *MSBM* = *MSWS* = *MSWM* = *MSE* = 0.

We therefore move on to the next term, r_i. Putting all other terms in (A2-1) equal to zero we get the following matrix:

r_1	r_1	r_1
r_2	r_2	r_2
r_3	r_3	r_3
$\bar{r}$	$\bar{r}$	$\bar{r}$

Here, $\bar{r}$ is the mean value of the three r_i. Again using Appendix 1, we get

$$MSBS = \frac{SSBS}{n-1} = \frac{\sum_{i,j}(S_i - \bar{x})^2}{n-1} = \frac{k \cdot \sum_i (S_i - \bar{x})^2}{n-1} = \frac{k \cdot \sum_i (r_i - \bar{r})^2}{n-1} \approx k \cdot \sigma_r^{\ 2}$$

(A2-3)

$$MSWM = \frac{SSWM}{k \cdot (n-1)} = \frac{\sum_{i,j}(x_{ij} - M_j)^2}{k \cdot (n-1)} = \frac{\sum_{i,j}(r_i - \bar{r})^2}{k \cdot (n-1)} = \frac{\sum_i (r_i - \bar{r})^2}{(n-1)} \approx \sigma_r^{\ 2}$$

where the "≈" sign means "is an estimate of". The last member in both equations follows when we realize that

$$\sum_i (r_i - \bar{r})^2 / (n-1)$$

is the square of the standard deviation of the three r_i values about their mean value $\bar{r}$; therefore, it is an estimate of the variance $\sigma_r^{\ 2}$. In fact, if this sampling of three r_i values from a normal distribution with variance $\sigma_r^{\ 2}$ is repeated a large number of times, then eq.(A2-3) means for example that the average value of *MSWM* will tend to be equal to $\sigma_r^{\ 2}$.

By similar procedures we may easily show that $MSBM = MSWS = 0$. From Appendix 1 we find the exact relation

$$MSE = \frac{k}{k-1} MSWM - \frac{1}{k-1} MSBS \qquad \text{(A2-4)}$$

Using (A2-3) in (A2-4), we find $MSE \approx 0$. In summary, therefore, the r_i term gives the contributions

$$\begin{aligned} MSBS &\approx k \cdot \sigma_r^{\ 2} \\ MSBM &= 0 \\ MSWS &= 0 \\ MSWM &\approx \sigma_r^{\ 2} \\ MSE &\approx 0 \end{aligned} \qquad \text{(A2-5)}$$

We next put all terms in (A2-1) except the c_j equal to zero. The resulting matrix is

c_1	c_2	$\bar{c}$
c_1	c_2	$\bar{c}$
c_1	c_2	$\bar{c}$
c_1	c_2	$\bar{c}$

Here, $\bar{c}$ is the mean value of the three c_j. We need not do all the above calculations again, but merely observe that rows and columns have changed roles. Thus, we will obtain the desired formulas simply by replacing k by n, σ_r by σ_c, *MSBS* by *MSBM*, *MSBM* by *MSBS*, *MSWS* by *MSWM* and *MSWM* by *MSWS*. For the MSE, we use again eq.(A2-5). We show *MSWS* explicitly:

$$MSWS = \frac{SSWS}{n \cdot (k-1)} = \frac{\sum_{i,j} (x_{ij} - S_i)^2}{n \cdot (k-1)} = \frac{\sum_{i,j} (c_j - \bar{c})^2}{n \cdot (k-1)} = \frac{\sum_{i} (c_j - \bar{c})^2}{(k-1)} \approx \sigma_c^{\ 2} \quad \text{(A2-6)}$$

where, again, the last member follows since we recognize the square of the standard deviation of the c_j about their mean value in the next last member. The result, i.e. the contributions from the c_j term, is therefore

$$\begin{aligned} MSBS &= 0 \\ MSBM &\approx n \cdot \sigma_c^{\ 2} \\ MSWS &\approx \sigma_c^{\ 2} \\ MSWM &= 0 \\ MSE &= 0 \end{aligned} \quad \text{(A2-7)}$$

Here we may insert the derivation in the case that the c_j terms are fixed, i.e. Model 3. The matrix looks precisely the same. However, in eq.(A2-6) we do not make the estimate ($\approx \sigma_c^{\ 2}$) in the last member, but simply use the fact that

$$MSWS = \frac{\sum_{j} (c_j - \bar{c})^2}{(k-1)} \equiv \theta_c^{\ 2} \quad \text{(A2-8)}$$

where the customary symbol θ_c^2 is defined. For Model 3 the contributions from the c_j term are therefore

$$\begin{aligned} MSBS &= 0 \\ MSBM &= n \cdot \theta_c^{\ 2} \\ MSWS &= \theta_c^{\ 2} \\ MSWM &= 0 \\ MSE &= 0 \end{aligned} \quad \text{(A2-9)}$$

We now consider the last term in (A2-1), i.e. we put all terms equal to zero except v_{ij}.The resulting matrix is given by

v_{11}	v_{12}	R_1
v_{21}	v_{22}	R_2
v_{31}	v_{32}	R_3
C_1	C_2	$\bar{v}$

Here R_i and C_j denote the row and column mean values. We may here observe that each matrix element v_{ij} is obtained by sampling from a normal distribution with variance $\sigma_v{}^2$. Moreover, each R_i value is the mean value of k = 2 matrix elements; its variance should therefore be $\sigma_v{}^2/k$. In the same way, each C_j value is the mean value of n = 3 matrix elements; its variance should therefore be $\sigma_v{}^2/n$.

From this we get

$$MSBS = \frac{SSBS}{n-1} = \frac{k \cdot \sum_i (S_i - \bar{x})^2}{n-1} = \frac{k \cdot \sum_i (R_i - \bar{v})^2}{n-1} \approx \sigma_v{}^2 \qquad \text{(A2-10)}$$

The last member follows if we observe that the next last member, apart from the factor k, is the square of the standard deviation of the R_i values about their mean value $\bar{v}$, i.e. it is an estimate of their variance:

$$\frac{\sum_i (R_i - \bar{v})^2}{n-1} \approx \sigma_v{}^2 / k \qquad \text{(A2-11)}$$

Proceeding in a similar manner to estimate *MSBM*, *MSWM* and *MSWS*, and using (A2-5) to estimate *MSE*, we find the following simple result for the contributions from the v_{ij} term:

$$\begin{aligned} MSBS &\approx \sigma_v{}^2 \\ MSBM &\approx \sigma_v{}^2 \\ MSWS &\approx \sigma_v{}^2 \\ MSWM &\approx \sigma_v{}^2 \\ MSE &\approx \sigma_v{}^2 \end{aligned} \qquad \text{(A2-12)}$$

For Model 2 we now find the total expression for the mean squares as estimates of the variances by adding the contributions (A2-5), (A2-7) and (A2-12). This gives

$$\begin{aligned} MSBS &\approx k \cdot \sigma_r{}^2 + \sigma_v{}^2 \\ MSBM &\approx n \cdot \sigma_c{}^2 + \sigma_v{}^2 \\ MSWS &\approx \sigma_c{}^2 + \sigma_v{}^2 \\ MSWM &\approx \sigma_r{}^2 + \sigma_v{}^2 \\ MSE &\approx \sigma_v{}^2 \end{aligned} \qquad \text{(A2-13)}$$

The estimates for Model 1 are obtained from (A2-13) simply by putting $\sigma_c{}^2 = 0$, and the estimates for Model 3 by replacing $\sigma_c{}^2$ with $\theta_c{}^2$, as shown by (A2-9).
