# Supplementary material for: Intraclass correlation – A discussion and demonstration of basic features
Source: PLoS One. 2019 Jul 22;14(7):e0219854. doi: 10.1371/journal.pone.0219854 (PMC6645485; doi:10.1371/journal.pone.0219854)
Supplement: S3 Appendix — Sampling from a normal distribution by means of the Monte Carlo method. (PDF) [file pone.0219854.s003.pdf]

Appendix 3
Sampling from a normal distribution by means of the Monte Carlo method

A normal distribution $G(x)$, also known as a "Gaussian", is, as well known, determined by its standard deviation σ and mean value μ. We wish to make a "random sampling" of an x value from this distribution.

To perform this sampling in practice, we use Monte Carlo simulation. This is a numerical method based on the use of so-called random numbers R. An algorithm (often called a "random number generator") produces a seemingly random sequence of numbers R between 0 and 1 (for example 0.5234, 0.1765, 0.8256, 0.0012 …). The exact sequence depends on the seed (or seeds), which may be an arbitrary integer (for example 2345 or 8645) used when calling the generator for the first time in a given simulation run. The presently used random number generator is a modified version of the subroutine RANECU described by F.James, in Computer Physics Communication 60 (1990), 329-344, adapted to give a single random number at each call.

In reality, the algorithm is deterministic, i.e. starting twice with the same seed (or seeds), you will get exactly the same sequence of numbers; nevertheless, it is such that you may assume that the probability of getting a particular number R is the same for all R values between 0 and 1. For example, you may in practice assume that the probability that a random number will have a value between 0.66 and 0.68 is 0.68 – 0.66 = 0.02.

By various mathematical tricks, the random number R distribution between 0 and 1 may be transformed into any desired probability distribution. For the sampling of values x from a normal distribution $G(x)$ we have used the so-called rejection method. For this we may start from an un-normalized and truncated Gaussian given by

$$\begin{aligned} G(x) &= \exp\left[-\frac{(x-\mu)^2}{2\sigma^2}\right] \qquad -4\sigma < x < +4\sigma \\ G(x) &= 0 \qquad\qquad x \le -4\sigma, \quad x \ge +4\sigma \end{aligned} \qquad \text{(A3-1)}$$

The truncation means that we make the approximation $G(x) = 0$ for x-values beyond four standard deviations from the mean. Note that the maximum value of $G(x)$ is = 1.

In order to sample an x-value we do as follows. First, call the random number generator and get an R value between 0 and 1, for the moment called R_1. Now, calculate an x-value given by

$$x = \mu - 4\sigma + R_1 \cdot 8\sigma \qquad \text{(A3-2)}$$

This x-value will evidently have a random position on the x-axis somewhere in the interval between μ-4σ and μ+4σ. For this x-value, calculate $g = G(x)$, using (A3-1). From eq.(A3-1) we can see that g will have a value between 0 and 1.

Next, call the random number generator again to get a second R value, for the moment called R_2. Now, R_2 may with equal probability have any value between 0 and 1. Consequently, the probability that the value of R_2 is somewhere in the interval between 0 and g will be precisely $g = G(x)$. If this is found to be the case, i.e. if $R_2 < g$, then the x value is "accepted", i.e. *used* as a sampled x value. If not, i.e. if $R_2 \ge g$, then the x value is "rejected".

The whole procedure is then repeated, i.e. started again by calling the random generator for a new R_1, until an "accepted" x value is obtained.

The probability that a randomly selected x value in the interval between $\mu - 4\sigma$ and $\mu + 4\sigma$ will be accepted is thus $g = G(x)$. If a large number of x values are generated in this way, they will be distributed in accordance with $G(x)$, i.e, distributed as if sampled from a normal distribution with mean value μ and standard deviation σ.
